# Tofacitinib repairs inflammation and mitochondrial dysregulation in GM-CSF-reprogrammed RA macrophages

**DOI:** 10.1038/s41423-026-01395-x

**Published:** 2026-03-04

**Authors:** Neha Satoeya, Stephanie R. Zack, Osama Al Zoubi, Sadiq Umar, Adel Burgos, Sara Abdulrab, Brian Zanotti, Michael V. Volin, Joseph A. Karam, Yinglin Xia, Diana C. Umali, Shiva Arami, Mina Al-Awqati, Huan T. Chang, Luke A. J. O’Neill, Georg Schett, Nadera Sweiss, Shiva Shahrara

**Affiliations:** 1 Jesse Brown VA Medical Center, Chicago, IL USA; 2 Department of Medicine, Division of Rheumatology, The University of Illinois Chicago, Chicago, IL USA; 3 Department of Microbiology and Immunology, Midwestern University, Downers Grove, IL USA; 4 Department of Orthopedic Surgery, University of Illinois College of Medicine, Chicago, IL USA; 5 School of Biochemistry and Immunology, Trinity Biomedical Sciences Institute, Trinity College, Dublin, Ireland; 6 Department of Internal Medicine Rheumatology and Immunology Friedrich-Alexander Universität Erlangen-Nürnberg and Universitätsklinikum Erlangen, Erlangen, Germany; 7 Deutsches Zentrum Immuntherapie, Friedrich-Alexander Universität Erlangen-Nürnberg and Universitätsklinikum Erlangen, Erlangen, Germany

**Keywords:** GM-CSF, Macrophages, NFIL3, Mitochondrial fragmentation, Oxidative stress, Autoimmunity, Predictive markers

## Abstract

Rheumatoid arthritis (RA) exhibits heterogeneous endotypes, complicating treatment strategies. GM-CSF and GM-CSFRα are enriched in RA synovial CD68⁺macrophages (MΦs), and are implicated in acute and chronic disease stages. Since anti-TNFi and anti-IL6R therapies did not effectively suppress GM-CSF/GM-CSFRα expression or the GM-CSF-associated landscape, we explored alternative therapeutic strategies to target GM-CSF function using RA blood, synovial tissues, and preclinical models. We demonstrate that GM-CSF-MΦs reprogrammed in RA blood and synovial tissue share a distinct IL1β⁺S100A⁺HIF1⁺IL10ˡᵒNFIL3/6ˡᵒ expression profile, manifested by mitochondrial oxidative stress and fragmentation. To correct the metabolic imbalance of GM-CSF-MΦs, cells were treated with a complex I inhibitor (i) or a glucose uptake blocker. Complex Ii did not broadly alter the inflammatory or metabolic networks or affect the mitochondrial dynamics remodeled by GM-CSF-MΦs. While the glucose uptake inhibitor (HK2i) reduced glycolysis-derived ATP, it had limited efficacy in restricting the inflammatory signature or restoring TCA enzymes in GM-CSF-MΦs. In contrast, tofacitinib achieved broad-spectrum effects by downregulating GM-CSFRα expression and inhibiting STAT5 signaling. Moreover, tofacitinib redirected RA blood and synovial IL1β⁺S100A⁺HIF1⁺IL10ˡᵒNFIL3/6ˡᵒMΦs into a regulatory phenotype, reversing oxidative stress and mitochondrial fragmentation. In preclinical models, local GM-CSF overexpression induced MΦ-directed joint inflammation and metabolic dysregulation. Consistently, Tofacitinib reversed GM-CSF-differentiated murine IL1β⁺HBEGF⁺HIF1⁺MΦs by impeding STAT5 signaling, correcting metabolic dysregulation, and repairing mitochondrial fragmentation. In conclusion, anti-TNFi, anti-IL6R, and metabolic-targeted therapies were largely ineffective in modifying GM-CSF-MΦ pathology. Conversely, tofacitinib deactivation of STAT5 attenuates GM-CSF-MΦ-triggered inflammation and mitochondrial malfunction by restoring regulatory markers and rebalancing oxidative phosphorylation in RA specimens and/or preclinical models.

## Introduction

Rheumatoid arthritis (RA) is an autoimmune disease in which synovial tissue MΦs are the primary producers of inflammatory cytokines [1–3]. RA synovial MΦ expansion is strongly linked to ultrasound-detected clinical and synovial pathology [4], and their frequency is 25-fold enriched during flare [5]. Consequently, *infiltrating* MΦs sense and respond to the RA synovial tissue’s high energy and oxygen demands. Thus, understanding how hypoxic RA MΦs experience metabolic changes and mitochondrial structure remodeling will unravel novel therapeutic strategies. Intriguingly, we found that granulocyte-macrophage colony-stimulating factor (GM-CSF) aligns the RA MΦ inflammatory profile with mitochondrial oxidative stress.

GM-CSF is elevated in RA synovial fluid [6] and blood [7], and its secretion originates from Th17 cells [8], IL7-polarized Th cells [9], and IL1β/TNF-stimulated RA fibroblast-like synoviocytes (FLS) [10], mimicking FAP^+^CD90^+^ RA synovial FLS [11]. GM-CSF operates through its heteromeric GM-CSFR, which comprises an α chain that is specific to GM-CSF and a β chain shared with the IL3 and IL5 receptors [12, 13].

RA flares were observed in individuals treated with GM-CSF; concurrently, it was identified as a RA risk gene in GWAS. GM-CSF is also associated with arthritic pain in part by activating the IRF4/CCL17 pathway in myeloid cells [14–16]. In parallel, GM-CSF knockout mice [17, 18] and neutralizing antibody (Ab) therapies [18, 19] attenuated arthritis in preclinical models by reducing inflammatory monokines. Correspondingly, GM-CSF^⁻/⁻^ mice exhibit lung surfactant accumulation, a phenotype that resembles human pulmonary alveolar proteinosis observed in patients treated with anti-GM-CSF therapies [20, 21]. Moreover, recent Phase III clinical studies suggest that anti-GM-CSF Ab therapy does not achieve an ACR20 response and is inferior to anti-IL6R Ab therapy [22]. Building on these findings, we aimed to elucidate the mechanism by which GM-CSF links RA inflammation to mitochondrial dysfunction and develop innovative strategies to reverse its effects. We document that TLR4 ligation amplifies the expression of GM-CSF and GM-CSFRα in myeloid cells. Regardless of clinical response, the GM-CSF and GM-CSFRα/β transcriptomes in RA synovial tissue remain unchanged by DMARDs, anti-TNFi therapy, or anti-IL6R Ab blockade. Importantly, plasma measurements confirm that DMARDs do not significantly affect GM-CSF protein levels, underscoring the persistence of GM-CSF-driven pathology [23]. In contrast, we demonstrate that tofacitinib corrects GM-CSF-driven RA MΦ immunopathology.

During RA inflammation, synovial MΦs cope with high oxygen demands by shifting from oxidative phosphorylation to glycolysis [2, 24]. ATP and oxygen decline in RA synovial tissues [25, 26] can disrupt the balance of TCA metabolites and enzymes or alter mitochondrial structure [27–29]. Corroborating these findings, glucose uptake inhibition mitigated collagen-induced arthritis (CIA) by intercepting the trafficking of F4/80^+^iNOS^+ ^MΦs, along with downregulating IRFs, cytokines, and glycolytic intermediates. Furthermore, the blockade of oxidative metabolites via SUCNR1 (succinate receptor, GPR91) [30] or HIF1α [31] knockout attenuates preclinical arthritis by impairing myeloid cell migration, pathology, or metabolic activity [32].

Recent transformative AMP I [33] & II [34] studies have compared the pathotypes, leukocyte-rich versus leukocyte-poor, and osteoarthritis (OA) synovium using single-cell RNA sequencing (scRNAseq), mass cytometry, and flow cytometry to identify MΦ subpopulations. These studies have unveiled unique MΦ subsets in crosstalk with stromal and other immune cells [33, 34]. Other pioneering scRNAseq studies using RA synovial tissues at various disease stages have defined 2 MΦ subpopulations with distinct transcriptomic signatures (MERTK^+^TREM^hi^ & MERTK^+^LYVE1^+^) enriched in remission [35]. Although the IL1β^+^HBEGF^+^S100A^+^MERTK^lo^ MΦ gene signature has been detected across multiple RA synovial pathotypes, including IL1β^+^(SC-M1) [33] and MERTK^lo^ S100A12^+^ subsets [35], their conception and mechanism of action are unknown. Here, we report for the first time that GM-CSF reprograms monocytes into a unique oxidatively stressed IL1β^+^S100A^+^HIF1^+^IL10^lo^NFIL3/6^lo^ MΦs exhibiting repressed TCA enzymatic activity accompanied by citrate, succinate, and HIF1α accumulation. Consequently, the reduced mitochondrial (mito)ATP in GM-CSF-MΦs is offset by increased glycoATP, reflected in the upregulation of GLUT1, HK2, and LDHA. RA MΦs reprogrammed by GM-CSF displayed STAT5 signaling, coinciding with mitochondrial oxidative stress and increased fragmentation. Intriguingly, tofacitinib counteracts STAT5 signaling, mitochondrial dysregulation, and fragmentation in GM-CSF-MΦs while fine-tuning mitoATP production over glycoATP. Conversely, glucose uptake or complex I inhibition reverses some but not all distinct features of GM-CSF-MΦ-driven inflammatory or bioenergetic reprogramming. Our data suggest that anti-TNFi, anti-IL6R Ab, or metabolic therapies cannot correct GM-CSF-MΦs. Distinctly, tofacitinib inhibition of STAT5 signaling disrupts GM-CSF-MΦ-driven inflammation and mitochondrial malfunction, thereby restoring regulatory pathways and oxidative repair networks in RA specimens and preclinical models.

## Results

### GM-CSF and its receptor are involved in early and established RA

**Fig. 1:**
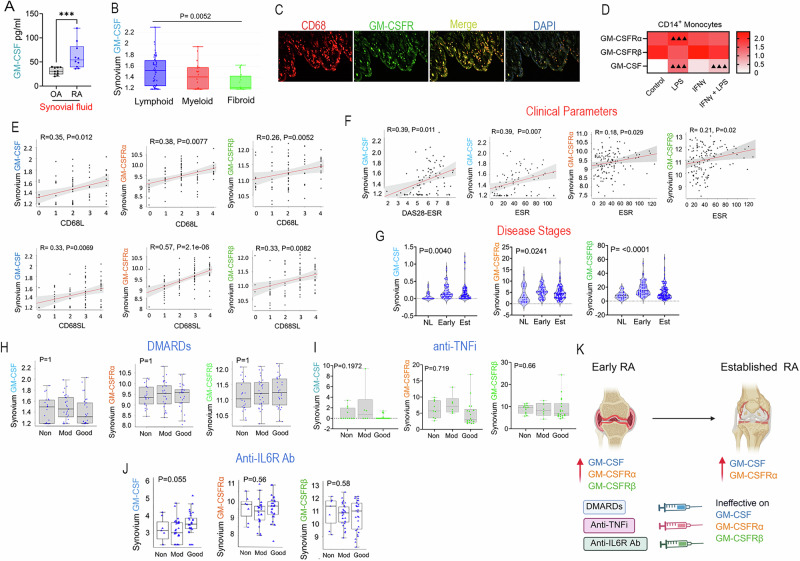
GM-CSF and its receptors correlate with RA clinical parameters and are unresponsive to DMARDs, anti-TNFi, and anti-IL6R Ab. **A** GM-CSF levels were measured in RA and OA synovial fluids by ELISA and analyzed via Mann–Whitney test, *n* = 10. **B** GM-CSF expression levels across histological pathotypes in RA synovial tissues were assessed by 1-way ANOVA with Bonferroni post-test, *n* = 90. **C** RA synovial tissue immunostaining displays GM-CSFR with CD68^+^MΦ co-localization (magx100) (Ab concentrations provided in the tables), *n* = 3. **D** Heatmap exhibits log-normalized counts of GM-CSF, GM-CSFRα, and GM-CSFRβ expression in CD14^+^ monocytes treated with LPS, IFNγ, or IFNγ + LPS compared to controls [69]. The Wald test determined statistical significance with adjustments for multiple testing, *n* = 3 (^▴▴▴^*P* < 0.001). **E**, **F** RA synovial tissue correlation of GM-CSF, GM-CSFRα, and GM-CSFRβ transcriptomes with histological CD68⁺MΦs (**E**, *n* = 90) [4] or DAS28-ESR and ESR (**F**, *n* = 81) [72] were analyzed using Spearman correlation with FDR adjustment. **G** Synovial GM-CSF, GM-CSFRα, and GM-CSFRβ transcriptomes in the normal (NL), early, and established (*n* = 23-93) RA synovial tissues were analyzed by a nonparametric Kruskal-Wallis test with Dunn’s correction for multiple comparisons [78]. **H** RA synovial tissue GM-CSF, GM-CSFRα, and GM-CSFRβ transcriptomes in non- (ΔDAS28 ≤ 0.6, *n* = 23), moderate- (ΔDAS28 ≤ 1.2 & >0.6, *n* = 29), or good- (ΔDAS28 > 1.2, *n* = 29) responders to DMARD therapies [4] were assessed by ANOVA with Bonferroni post-test. **I** RA synovial tissue GM-CSF, GM-CSFRα, and GM-CSFRβ transcriptomes stratified by non-, moderate-, or good-responders to anti-TNFi therapy (Etanercept, *n* = 46) [70] were analyzed by a nonparametric Kruskal–Wallis test with Dunn’s correction for multiple comparisons. **J** RA synovial tissue GM-CSF, GM-CSFRα, and GM-CSFRβ transcriptomes stratified by different responses to anti-IL6R Ab (Tocilizumab, *n* = 81) [71] were analyzed by the Wilcoxon signed-rank test. **K** Schematic illustrating that DMARDs, anti-TNFi, and anti-IL6R Ab do not impact RA GM-CSF or GM-CSFRα/β expression. All graphs display mean ± SEM. *p < 0.05, **p < 0.01, ***p < 0.001, and ****p < 0.0001

Initially, studies were conducted to determine the origin of RA GM-CSF-MΦs and their relationship with RA clinical parameters. We found that GM-CSF is enriched in RA compared to OA synovial fluids (Fig. 1A). The GM-CSF transcriptome is markedly elevated in synovial lymphoid cells compared to fibroid cells (Fig. 1B). Meanwhile, GM-CSFR staining is enhanced in RA synovial tissue MΦs compared to normal controls (NL) (Fig. 1C, Suppl-1A). GM-CSF and GM-CSFRα, but not GM-CSFRβ, are upregulated in myeloid cells in response to LPS (Fig. 1D). In keeping with the importance of GM-CSF and GM-CSFRα/β in RA pathophysiology, their expression is closely linked to CD68⁺MΦs in the synovial lining (L) and sublining (SL, Fig. 1E). Synovial GM-CSF transcription is associated with DAS28-ESR (Disease Activity Score 28-joint count using Erythrocyte Sedimentation Rate) and ESR, while GM-CSFRα/β levels are reflective of patient ESR values (Fig. 1F). We found that synovial GM-CSF and GM-CSFRα are expressed in early and established RA (Fig. 1G). Interestingly, regardless of clinical response (Non, Moderate, or Good), RA standard-of-care therapies, including DMARDs, anti-TNFi, and anti-IL6R Ab, were ineffective on GM-CSF and GM-CSFRα/β transcriptomes (Fig. 1H–J). Overall, GM-CSF and GM-CSFRα/β could have a positive correlation with clinical parameters and are involved in the acute and/or chronic phases of RA MΦ reprogramming (Fig. 1K).

### GM-CSF reprograms RA monocytes into IL1β^+^HBEGF^+^S100A12^+^MERTK^lo^NUPR1^lo^NFIL3/6^lo^ macrophages (GM-MΦs)

**Fig. 2:**
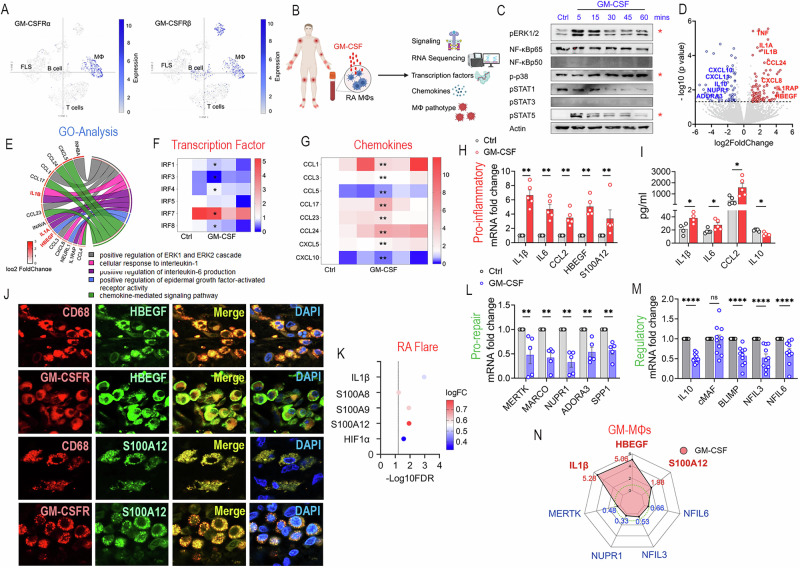
RA GM-MΦs display distinct signaling and inflammatory profiles. **A** scRNAseq of RA synovial tissue identified MΦ subpopulations with distinct GM-CSFRα/β expression, *n* = 18 [33]. **B** Our experimental workflow of RA GM-MΦs includes activated signaling pathways, differential gene profiling of TFs, chemokines, and MΦ pathotype characterization. **C** Myeloid cells were treated with GM-CSF (100 ng/ml, 0-60 min), and pERK, p65, p50, p-p38, pSTAT1, pSTAT3, and pSTAT5 were normalized to equal actin loading, *n* = 3 (**C**, raw data Suppl-4-1). **D**, **E** Differentially expressed genes following treatment with GM-CSF (100 ng/ml) for 12 h, compared to control, were analyzed by RNAseq and visualized as a volcano plot (**D**, *n* = 3) along with GO analysis (**E**, *n* = 3). Genes with significant upregulation (*p* < 0.05, Log2FC ≥ 1.0) are highlighted in red, while significantly downregulated genes (*p* < 0.05, Log2FC ≤ -1.0) are shown in blue. **F–K** RA MΦs were untreated or treated with GM-CSF (100 ng/ml) for 6 h before TFs (**F**, *n* = 4, Suppl-1C), chemokines (**G**, *n* = 5, Suppl-1D), and monokines (**H**, *n* = 5) were quantified by qRT-PCR. **I**. RA MΦs were untreated or treated with GM-CSF (100 ng/ml, 24 h), and IL1β, IL6, CCL2, and IL10 protein levels were quantified by ELISA, *n* = 4–5. **J**. Co-localization of HBEGF and S100A12 on CD68^+^MΦs or GM-CSFR^+^ cells was visualized by IF in RA synovial tissues (magx400), *n* = 3. **K** GM-MΦ-associated genes are upregulated during disease flare in RA PBMCs above baseline (*n* = 46, FDR-adjusted *p* < 0.05) [5]. Prorepair (**L**, *n* = 5) and regulatory (**M**, *n* = 10) genes are shown in GM-MΦs (100 ng/ml, 6 h) by qRT-PCR. **N** The GM-MΦ profile is visualized by a radar plot, which summarizes findings in (**H**, **L**, **M**). Data are presented as mean ± SEM; statistical differences were determined by the Mann–Whitney test: *p < 0.05, **p < 0.01, ***p < 0.001, and ****p < 0.0001

To explore the responsiveness of RA synoviocytes to GM-CSF, GM-CSFRα/β expression was interrogated by scRNAseq. Distinct from GM-CSF expression (Fig. 1B), GM-CSFRα/β expression clustered in RA MΦs and was absent in B or T cells and FLS, indicating that RA MΦs are the primary responders to GM-CSF (Fig. 2A). Myeloid cells stimulated with GM-CSF displayed ERK, p38, and STAT5 signaling (Fig. 2B,C). Conversely, p50/p65 NF-κB, STAT1/3, and JAK1/3 remained inactive (Fig. 2C, Suppl-1B, Suppl-4-1, Suppl-4-5). RNAseq analysis of GM-MΦs uncovered that upregulation of unique pro-inflammatory gene signatures (ERK1/2, IL1β, IL6, EGFR), coincides with downregulation of pro-repair pathways (NUPR1, ADORA3, IL10) (Fig. 2D,E). Reprogramming of GM-CSF-MΦs (GM-MΦs) was associated with a particular transcription factor (TF), IRF7, while IRF1/3/4/8 were reduced (Fig. 2F, Suppl-1C). In parallel, specific monocyte and T cell chemokines, CCL1, CCL3, CCL17, CCL23, CCL24, and CXCL5, were amplified in GM-MΦs, whereas the proangiogenic chemokines CXCL10 and CCL5 were suppressed (Fig. 2G, Suppl-1D). GM-MΦs revealed a unique transcriptional profile with enriched IL1β, HBEGF, and S100A12 (Figs. 2H, N). IL1β, IL6, CCL2 expansion, and IL10 reduction were substantiated in GM-MΦs by ELISA (Fig. 2I). Moreover, the IF studies established that RA synovial tissue CD68^+^GM-CSFR^+^MΦs coexpress HBEGF and S100A12 (Fig. 2J). Extending these findings, circulating levels of IL1β, S100A8/9/12, and HIF1α were elevated during RA flare, highlighting their significance in disease pathology (Fig. 2K). Concurrently, remission-associated signature genes, including MERTK, MARCO, NUPR1, ADORA3, and SPP1, were restrained in GM-MΦs (Fig. 2L). In parallel, regulatory genes, IL10, BLIMP, NFIL3, and NFIL6, but not cMAF transcription, were downregulated in GM-MΦs (Fig. 2M). Taken together, monocytes reprogrammed by GM-CSF display a specific signature (IL1β^+^HBEGF^+^S100A12^+^MERTK^lo^NUPR1^lo^NFIL3/6^lo^) found in RA with active disease (Fig. 2N).

### GM-MΦs shift their metabolic activity from mitoATP to glycoATP

**Fig. 3:**
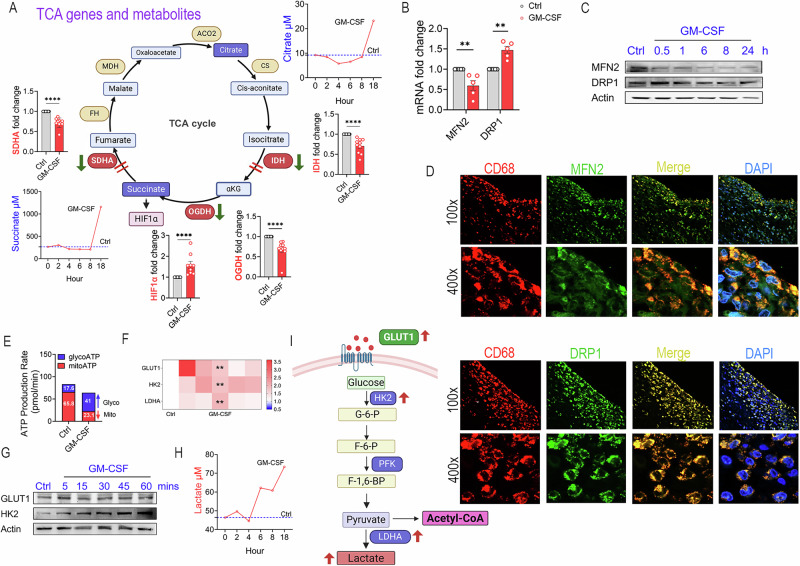
GM-CSF-differentiated RA MΦs exhibit a hyperglycolytic profile. **A** RA MΦs were untreated or treated with GM-CSF (100 ng/ml), and TCA enzymes (IDH, OGDH, SDHA) and HIF1α (6 h) transcription or TCA metabolite levels (citrate, succinate, 0-18 h) were quantified by qRT-PCR (*n* = 10) or colorimetric assay (representative of *n* = 3–5). **B** RA MΦs were untreated or treated with GM-CSF (100 ng/ml) for 6 h, and transcript levels of MFN2 and DRP1 were determined by qRT-PCR, *n* = 5. **C** Myeloid cells were treated with GM-CSF (100 ng/ml) for 0-24 h before detecting MFN2 and DRP1 by western blotting, *n* = 3 (Raw WBs: Suppl-4-2). **D** In RA synovial tissue CD68^+^MΦs, expansion of the DRP1-MFN2 ratio suggests mitochondrial fragmentation as displayed by IF immunostaining (*n* = 3, magx100 and magx400). **E**, **F** RA MΦs were untreated or treated with GM-CSF (1μg/ml). MitoATP and glycoATP production rates were measured by Seahorse XF Real-time ATP rate assay (**E**), and GLUT1, HK2, and LDHA transcription levels were quantified by qRT-PCR, *n* = 5 (**F**). **G **Myeloid cells were treated with GM-CSF (100 ng/ml) for 0–60 min before detection of GLUT1 and HK2 by western blot analysis, *n* = 3 (Raw WBs: Suppl-4-3). **H** Representative lactate levels are shown in RA MΦ conditioned media, untreated or treated with GM-CSF (100 ng/ml, 0-18 h), *n* = 7. **I** Diagram summarizing GM-CSF-driven glycolytic reprogramming. Data are presented as mean ± SEM; statistical analysis was performed using the Mann–Whitney test: *p < 0.05, **p < 0.01, ***p < 0.001, and ****p *<* 0.0001

We next delineated how bioenergetics and mitochondrial dynamics were impacted by GM-MΦ rewiring. RA GM-MΦ reprogramming coincided with TCA enzyme repression of IDH, OGDH, and SDHA; however, ACO2, CS, FH, and MDH2 remained unchanged (Fig. 3A, Suppl-1E). The breakdown of specific TCA enzymes (IDH, SDHA) results in citrate, succinate, and HIF1α buildup (Fig. 3A) coupled with the transcriptional imbalance of fission-associated protein, DRP1 (Dynamin-related protein 1), over fusion marker, MFN2 (Mitofusin 2, Fig. 3B). In GM-MΦs, DRP1 expression was upregulated, whereas MFN2 protein levels were diminished over time (Fig. 3C, Suppl-4-2). Corroborating these findings, in RA synovial tissues, the frequency of CD68^+^DRP1^+^MΦs was higher than CD68^+^MFN2^+^MΦs, indicating an overbalance of fission over fusion (Fig. 3D). The dysfunctional Krebs cycle in GM-MΦs switches its biogenesis from mitoATP (65% to 23%) to glycoATP (17% to 41%) (Fig. 3E). Along with this bioenergetic shift, GLUT1, HK2, LDHA transcriptional and protein levels, as well as lactate buildup, were amplified in GM-MΦs (Fig. 3F-I, Suppl-4-3). Overall, our data display a metabolic shift towards glycolysis and mitochondrial fragmentation in RA GM-MΦs.

### Blockade of glucose uptake or Complex I inhibition does not fully repair RA GM-MΦs

**Fig. 4:**
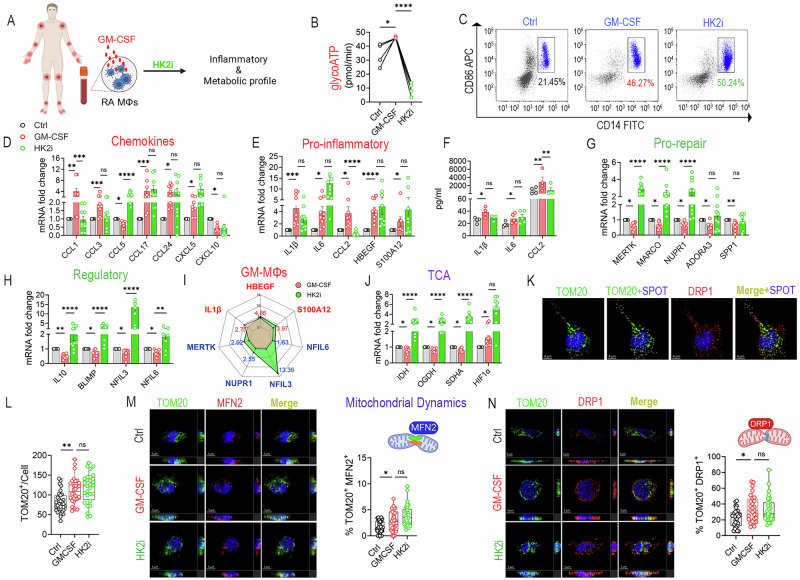
HK2 inhibition partially restores RA GM-MΦs. **A** Schematic representation of studies defining the impact of HK2i therapy on the inflammatory and metabolic networks triggered by GM-MΦs. **B** RA MΦs were untreated or treated with GM-CSF (1 μg/ml) ± HK2i (50 mM), and glycoATP rate was measured by Seahorse XF Real-time ATP rate assay, *n *= 4. **C** RA MΦs were untreated or treated withl GM-CSF (100 ng/ml) ± HK2i (5 mM) for 24 h, followed by flow cytometry analysis of %CD14⁺CD86⁺MΦs,* n* = 5 (gating strategy, Suppl-2A). **D–H**. RA MΦs were untreated or treated with GM-CSF (100 ng/ml) ± HK2i (5 mM) for 6 h before quantifying chemokines **(D)**, pro-inflammatory **(E)**, pro-repair **(G)**, and regulatory **(H)** transcriptomes by qRT-PCR, *n* = 5–10. **F** RA MΦs were untreated or treated with GM-CSF (100 ng/ml) ± HK2i (5 mM) for 24 h before IL1β, IL6, and CCL2 levels were measured by ELISA, *n* = 4–5. **I** The impact of HK2i on the GM-MΦ signature is visualized by a radar plot summarizing findings in (**D**-**H**). **J** TCA enzymes and intermediates were quantified in RA MΦs treated with GM-CSF (100 ng/ml) ± HK2i (5 mM) for 6 h by qRT-PCR, *n* = 10. **K** Representative image of RA MΦs stained for TOM20. Imaris was used to build a spots algorithm around the TOM20 signal. **L** RA MΦs were untreated or treated with GM-CSF ± HK2i. Box plots show the number of TOM20^+^/cell that were quantified by Imaris, *n* = 30. The %TOM20^+^MFN2^+^ (**M**) or %TOM20^+^DRP1^+^ (**N**) spots per cell were quantified in 30 cells by Imaris. Data are presented as mean ± SEM. Significant differences were determined by the nonparametric Kruskal–Wallis test followed by Dunn’s multiple comparisons test: *p < 0.05, **p < 0.01, ***p < 0.001, and ****p < 0.0001

**Fig. 5:**
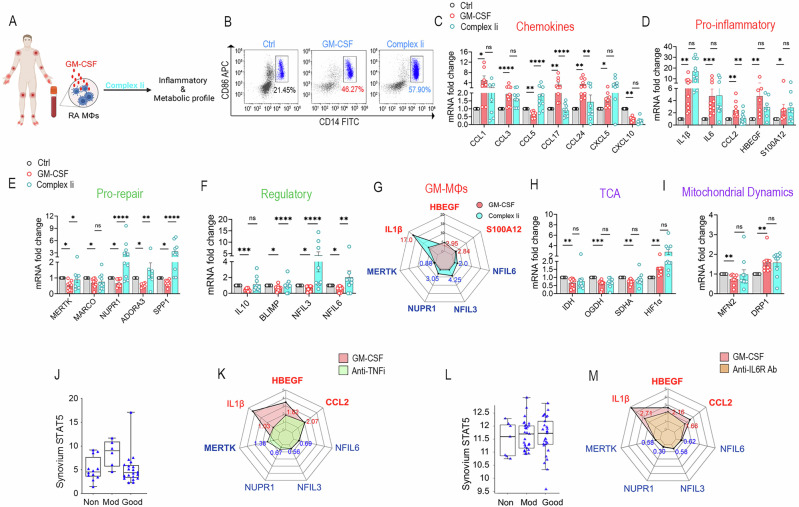
Complex Ii fails to reverse RA GM-MΦ pathology. **A** Schematic representation of studies examining the impact of the Complex I inhibitor on the inflammatory and metabolic landscapes of GM-CSF-differentiated MΦs. **B** RA MΦs were treated with GM-CSF (100 ng/ml) ± Complex Ii (100 nM) for 24 h before flow cytometry analysis of %CD14⁺CD86⁺MΦs, *n* = 5. **C**–**I** RA MΦs were untreated or treated with GM-CSF (100 ng/ml) ± Complex Ii (100 nM) for 6 h before chemokines (**C**), pro-inflammatory (**D**), pro-repair (**E**), regulatory (**F**) genes or TCA intermediates (**H**), and mitochondrial dynamics regulators (DRP1/MFN2) (**I**) transcription levels were quantified by qRT-PCR, *n* = 5–10. **G** The effect of Complex Ii on the GM-MΦ signature is visualized by a radar plot summarizing findings in **C**-**F**. RA synovial tissue STAT5 transcriptomes stratified by non-, moderate-, or good-responders to anti-TNFi (Etanercept, *n* = 46) [70] (**J**), or anti-IL6R Ab (Tocilizumab, *n* = 81) [71] therapies (**L**). The impact of anti-TNFi (10 μg/ml, **K**) or anti-IL6R Ab (10 μg/ml, **M**) on GM-MΦ signatures is shown in a radar plot summarizing pro-inflammatory, pro-repair, and regulatory profiles measured by qRT-PCR (*n* = 5–6, Suppl-2C, D). Data are presented as mean ± SEM. Significant differences were determined by the nonparametric Kruskal–Wallis test followed by Dunn’s multiple comparisons test: *p < 0.05, **p < 0.01, ***p < 0.001, and ****p < 0.0001

Given that the oxidatively stressed GM-MΦs are hyperglycolytic, we investigated whether inhibiting HK2 or Complex I would correct the inflammatory or metabolic profiles and alter mitochondrial dynamics. We established that the HK2 inhibitor (i) blocked GM-MΦ-induced glycoATP production but did not alter CD14⁺CD86⁺MΦ polarization (Fig. 4A-C, Suppl-2A). HK2i fine-tuning of GM-MΦs was restricted mainly to transcriptional regulation of CCL1, CCL5, CCL2, MERTK, MARCO, NUPR1, IL10, BLIMP, and NFIL3/6, along with partial restoration of TCA enzymes IDH, OGDH, and SDHA (Fig. 4D-J). We confirmed that the effects of HK2i on prorepair mediators occur independently of cell survival (Suppl-2B). Nonetheless, the heightened inflammatory and metabolic signatures, characterized by elevated levels of IL1β, IL6, HBEGF, S100A12, CCL3, CCL17, CCL24, CXCL5, and HIF1α, along with the diminished pro-repair markers ADORA3 and SPP1, remained unaffected by HK2i treatment (Fig. 4D–J). Moreover, the increase in the number of mitochondria/cell and the 10-fold DRP1/MFN2 ratio expansion was unchanged in the HK2i group compared to control-treated GM-MΦs (Fig. 4K–N). Similar to HK2i, the complex Ii (IACS-010759) was ineffective on the GM-MΦ-driven CD14⁺CD86⁺MΦ reprogramming and had only a limited impact on the expression of CCL2, CCL17/24, MERTK, NUPR1, ADORA3, SPP1, BLIMP, and NFIL3/6 (Fig. 5A–G). Additionally, TCA enzymes and mitochondrial dynamics were not restored by Complex Ii (Fig. 5H, I). Altogether, HK2i and Complex Ii have a partial rehabilitative effect on GM-MΦ-induced pathogenesis.

### Unlike tofacitinib, anti-TNFi and anti-IL6R antibodies exert modest effects on GM-MΦs

Next, experiments were designed to evaluate the efficacy of the standard-of-care RA therapies on GM-MΦs. Anti-TNFi (Humira) and anti-IL6R Ab (tocilizumab) therapies reduced IL1β transcription in GM-MΦs; however, only anti-TNFi restored MERTK, MARCO and ADORA3 expression in these cells (Fig. 5K, M, Suppl-2C, D). This limited activity aligns with their lack of impact on STAT5 expression in RA synovial tissue across all patient groups (Fig. 5J, L). Consistently, independent studies in RA patients show that anti-TNFi or anti-IL6R Ab therapy does not alter synovial GM-CSF expression or its receptor subunits (GM-CSFRα/β), even among clinical responders (Fig. 1I, J), further supporting this interpretation.

**Fig. 6:**
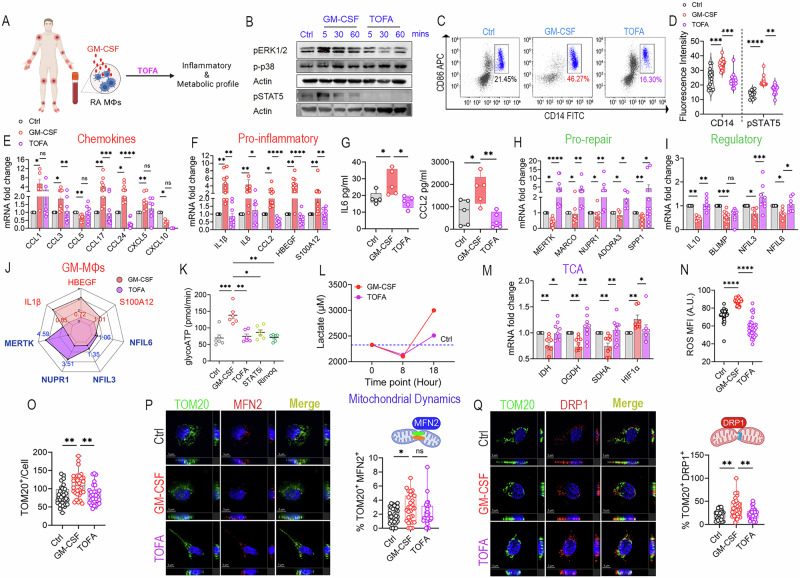
Tofacitinib counteracts the RA GM-MΦ pathotype. **A** Schematic representation of studies examining the role of tofacitinib (TOFA, 10 μM) on the inflammatory and metabolic profiles of GM-MΦs. **B** Myeloid cells treated with GM-CSF (100 ng/ml) ± TOFA (10 μM) for 0–60 min were assessed for ERK, p38, and STAT5 activation by western blot analysis, *n* = 3 (Suppl-4-4). **C** RA MΦs were untreated or treated with GM-CSF (100 ng/ml) ± TOFA (10 μM) for 24 h before flow cytometry analysis of %CD14⁺CD86⁺MΦs, *n* = 5. **D** Myeloid cells were untreated or treated with GM-CSF ± TOFA (10 μM) for 1 h before CD14 and pSTAT5 immunostaining was quantified by ImageJ, *n* = 15. **E–I** RA MΦs were untreated or treated with GM-CSF (100 ng/ml) ± TOFA (10 μM) for 6 h before chemokines (**E**), pro-inflammatory (**F**), pro-repair (**H**), and regulatory (**I**) transcriptomes were quantified by qRT-PCR, *n* = 5–10. **G** RA MΦs were untreated or treated with GM-CSF ± TOFA for 24 h, and CCL2 and IL6 protein levels were quantified using ELISA, *n* = 5. **J** The effect of tofacitinib on the GM-MΦ signature is visualized by a radar plot summarizing findings in (**E**–**I**). **K** RA MΦs were untreated or treated with GM-CSF (1 μg/ml) ± TOFA (100 μM), STAT5i (100 μM), or Rinvoq (100 μM), before glycoATP was measured using Seahorse XF ATP rate assay, *n* = 6. **L** RA MΦs were untreated or treated with GM-CSF ± TOFA for 0-18 h before measuring lactate colorimetrically, *n* = 5–8. **M** RA MΦs were untreated or treated with GM-CSF ± TOFA for 6 h, and the transcript level of TCA enzymes and HIF1α was quantified using qRT-PCR, *n* = 6-10. **N** ROS was determined in control, GM-MΦs ± TOFA (4 h) by confocal microscopy using DCFDA dye, and images were captured by ImageJ and presented as MFI in individual cells, *n* = 33. **O**–**Q** RA MΦs were untreated or treated with GM-CSF ± TOFA. Box plots visualize the number of TOM20^+^/cell (**O**), *n* = 30. The %TOM20^+^MFN2^+^ (**P**) or %TOM20^+^DRP1^+^ spots per cell **(Q)** was quantified in 30 cells by Imaris. Data are presented as mean ± SEM; significant differences between multiple groups were determined by the nonparametric Kruskal-Wallis test followed by Dunn’s multiple comparisons test: *p < 0.05, **p < 0.01, ***p < 0.001, and ****p < 0.0001

Distinctly, tofacitinib impaired CD14⁺CD86⁺MΦ reconfiguration and pSTAT5 fluorescence intensity in CD14^+ ^myeloid cells without affecting p-p38 (Fig. 6A–D, Suppl-4-4). In GM-MΦs, transcription of the highly elevated chemokines CCL3, CCL17, and CCL24 was downregulated in response to tofacitinib (Fig. 6E). Accordingly, tofacitinib redirected the GM-MΦ profile from a pro-inflammatory (IL1β, HBEGF, S100A12, CCL2, and IL6) to a pro-repair (MERTK, MARCO, NUPR1, ADORA3, and SPP1) signature (Fig. 6F–H). Similarly, in GM-MΦs, the balance was shifted to regulatory genes (IL10, NFIL3/6) and away from the inflammatory signature (Fig. 6I, J). Furthermore, tofacitinib repairs oxidatively stressed GM-MΦs by restructuring glycoATP to mitoATP (Fig. 6K, Suppl-2E), as evidenced by the expansion of IDH, OGDH, and SDHA, as well as the suppression of ROS, HIF1α, and lactate (Fig. 6L–N). The inhibitory effect of Tofacitinib on glycoATP was independently recapitulated by pharmacologic STAT5 inhibition and by treatment with Rinvoq (Fig. 6K). Notably, Rinvoq was also associated with a marked resolution of the GM-MΦ inflammatory phenotype, further reinforcing a shared and mechanistically conserved role for JAK/STAT signaling in driving the metabolic and inflammatory programs of GM-MΦs (Suppl-2F). Tofacitinib also fine-tuned GM-MΦ-mediated oxidative stress by reducing mitochondria/cell frequency, along with mitochondrial DRP1 positivity, to baseline levels, while MFN2 remained unaffected (Fig. 6O–Q). Collectively, Tofacitinib extensively repairs the inflammatory and metabolic landscapes of GM-MΦs by remodeling them into regulatory MΦs. As with newer JAK1 inhibitors such as Rinvoq, Tofacitinib targets a broader spectrum of JAK/STAT pathways, including STAT5 signaling in GM-MΦs.

### Tofacitinib intercepts GM-CSF-mediated RA synovial tissue inflammation and oxidative stress

**Fig. 7:**
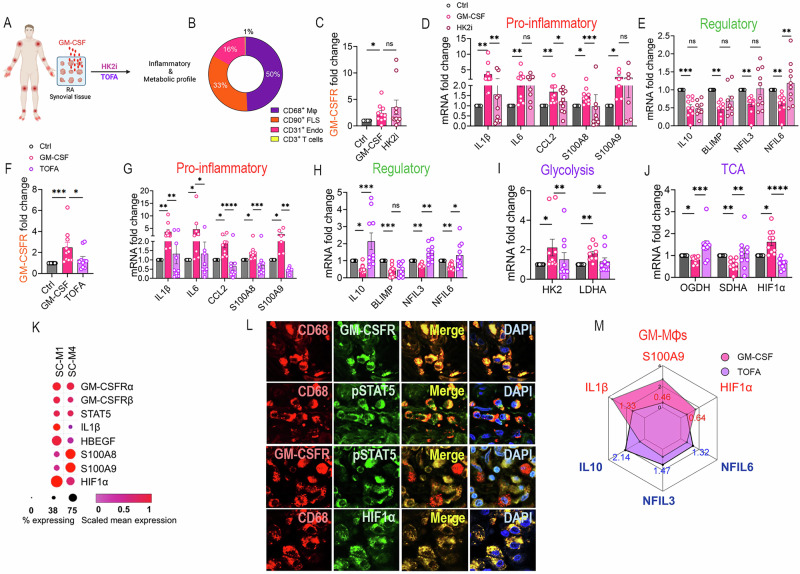
Tofacitinib counteracts GM-CSF-mediated RA synovial tissue inflammation and oxidative stress. **A** Schematic representation compares the impact of HK2i with TOFA on the GM-CSF profile in RA synovial tissues. **B** RA synovial tissues were pathotyped by quantifying CD68⁺MΦs, CD90⁺FLS, CD31⁺endothelial cells, and CD3⁺T cells to determine myeloid enrichment [79], *n* = 5. **C–E** RA synovial tissues were untreated or treated with GM-CSF (200 ng/ml) ± HK2i (10 mM, 6 h), and GM-CSFR (**C**, *n* = 10), pro-inflammatory (**D**, *n* = 10), and regulatory genes (**E**, *n* = 10) were quantified via qRT-PCR. **F–J** RA synovial tissues were untreated or treated with GM-CSF (200 ng/ml) ± TOFA (20 μM) for 6 h before GM-CSFR (**F**, *n* = 10), pro-inflammatory (**G**, *n* = 6-10), regulatory (**H**, *n* = 10), glycolytic genes (**I**, *n* = 10), and TCA intermediates (**J**, *n* = 10**)** were detected by qRT-PCR. **K** GM-CSFRα/β and their signature genes cluster in distinct RA synovial tissue MΦ subpopulations [33], *n* = 18. **L** RA synovial tissues were immunostained to authenticate the co-localization of GM-CSFR, pSTAT5, HIF1α, and CD68^+^MΦs (*n* = 3, magx400). **M** The effect of tofacitinab on the GM-CSF signature in RA synovial tissues is visualized by a radar plot summarizing findings in (**G**–**J**). Data are presented as mean ± SEM; significant differences were determined by the nonparametric Kruskal-Wallis test with Dunn’s multiple comparisons test: *p < 0.05, **p < 0.01, ***p < 0.001, and ****p < 0.0001

Prescreened RA synovial tissues were predominantly of the myeloid-rich pathotype (50%), compared with FLS, endothelial cells, and T cells, which accounted for 33%, 16%, and 1%, respectively (Fig. 7A, B). We noted that the RA synovium integrity was disrupted by digestion; therefore, all studies were performed using RA synovial tissue cultures. Tofacitinib, but not HK2i, downregulated synovial GM-CSFR expression in response to GM-CSF (Fig. 7C, F). Supporting this notion, the HK2i effect on the GM-CSF profile in RA synovium was limited to reducing IL1β, CCL2, and S100A8, elevating NFIL6 levels (Fig. 7D, E).

GM-CSF-induced transcription of IL1β, IL6, CCL2, S100A8/9, HK2, LDHA, and HIF1α was diminished by tofacitinib in RA synovial tissues (Fig. 7G–J). In parallel, tofacitinib rescued the GM-CSF-mediated repression of IL10, NFIL3/6, OGDH, and SDHA transcriptomes in RA synoviocytes (Fig. 7H, J). In RA synovial tissue M1(IL1β^+^)- and M4(IFN^+^)-MΦ subtypes, GM-CSFRα/β are colocalized with elevated STAT5,IL1β, HBEGF, S100A8/9, and HIF1α expression (Fig. 7K). Histological findings substantiated the coexpression of GM-CSFRα, pSTAT5, HIF1α, HBEGF, and S100 in RA synovial CD68^+^MΦs (Fig. 7L and 2J). Intrigingly, GM-CSF-cultivated conditioned media from RA MΦs failed to induce fibrokines, IL6, and CCL2 in RA FLS, indicating that neither GM-CSF nor RA MΦ-derived conditioned media are potent drivers of RA FLS activation (Suppl-2G). In short, our data suggest that in RA synovial tissues, tofacitinib can correct the GM-CSF-induced inflammatory MΦ signature (IL1β, HIF1α, S100) by escalating the regulatory profile (IL-10, NFIL3/6) and transitioning the metabolism towards a homeostatic phenotype (Fig. 7M). As a proof of concept, the effectiveness of tofacitinib was tested in preclinical arthritis models.

### Tofacitinib mitigates GM-CSF-induced arthritis

**Fig. 8:**
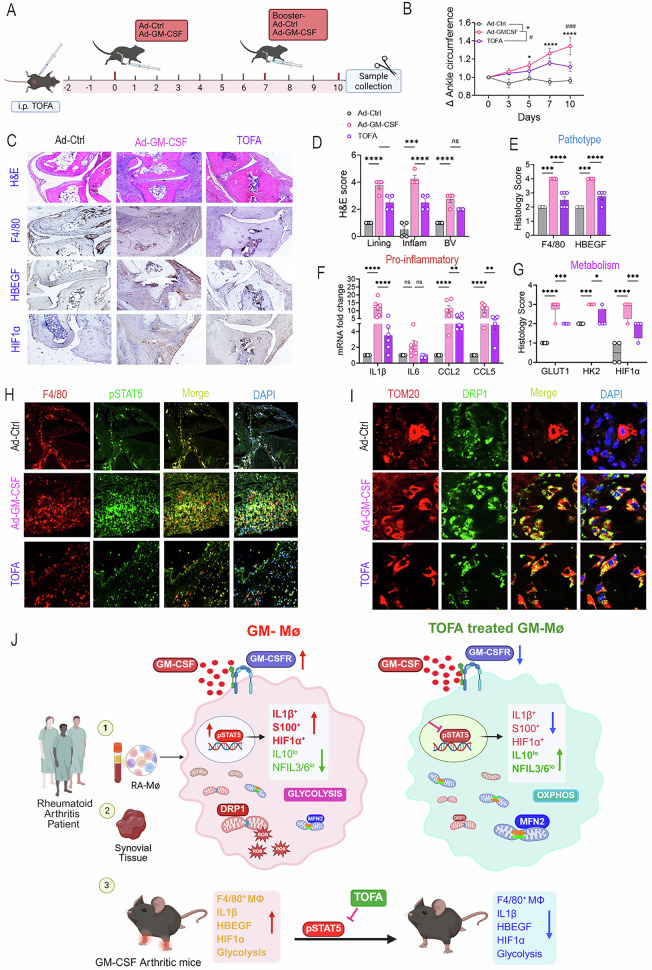
Tofacitinib therapy mitigates GM-CSF-induced arthritis by resolving the inflammatory and glycolytic imprints. Schematic visualization **(A)** and Δ ankle circumference **(B)** of mice that received i.a. injection of Ad-Control or Ad-GM-CSF (days 0 and 7), and the arthritic group received TOFA (10 mg/kg) daily over 10 days, *n* = 10 ankles in 5 mice. **C** Ankles from Ad-Ctrl and Ad-GM-CSF ± TOFA were stained for H&E, F4/80, HBEGF, and HIF1α. Joint lining thickness, inflammation, and blood vessel formation (**D**), F4/80 and HBEGF (**C,** **E**), GLUT1, HK2, and HIF1α (**C,** **G**) staining were scored on a 0-5 scale [79], *n* = 4. **F** IL1β, IL6, CCL2, and CCL5 transcriptional regulation was determined by qRT-PCR, *n* = 6. **H**, **I** Ankles were stained for colocalization of F4/80 and pSTAT5 (magx100) **(H)** or DRP1 and TOM20 (**I**) (magx400), *n* = 3. **J** The schematic figure summarizes the mechanism by which tofacitinib counteracts shared RA blood and ST IL1β⁺S100A⁺HIF1⁺IL10^lo^NFIL3/6^lo^ MΦs and experimental arthritis through STAT5 deactivation. Data are presented as mean ± SEM, and statistical differences were determined by a 2-way ANOVA test with Tukey’s method for adjusting for multiple comparisons: *p < 0.05, **p < 0.01, ***p < 0.001, and ****p < 0.0001

To evaluate the effect of tofacitinib on GM-CSF-induced arthritis, wild-type (WT) mice were intra-articularly injected with adenovirus control (Ad-Ctrl) or Ad-GM-CSF on days 0 and 7. Mice in the Ad-GM-CSF group received daily intraperitoneal tofacitinib, and ankles were harvested on day 10 (Fig. 8A). Local injection of GM-CSF progressively advanced joint swelling, which was mitigated by tofacitinib on day 10 post-onset (Fig. 8B). Moreover, GM-CSF-induced joint lining thickness, inflammation, and F4/80⁺ and HBEGF⁺ MΦ frequencies were restrained by tofacitinib therapy (Fig. 8C–E). Like reprogrammed GM-MΦs in RA blood and synovial tissues, the preclinical joint inflammatory profile (IL1β, CCL2, CCL5) and STAT5 signaling associated with local GM-CSF expression were resolved in response to tofacitinib (Fig. 8F, H). In tandem, tofacitinib therapy abolished GM-CSF-driven hypoxic (HIF1α) and glycolytic (GLUT1/HK2) intermediates, promoting oxidative repair (Fig. 8C, G). Tofacitinib’s ability to restore pro-repair factor (IL-10) and TCA cycle enzymes (IDH, OGDH, SDHA) in RA blood GM-MΦs and synovial tissue-reprogrammed MΦs was not replicated in GM-CSF-induced arthritis (Suppl-3A, B). Unlike TOM20⁺MFN2⁺ cells, which remained unchanged across all treatment groups, the elevated TOM20⁺DRP1⁺ cell levels in GM-CSF-induced arthritic mice were reduced by tofacitinib (Fig. 8I, Suppl-3C). Extending these findings, therapeutic tofacitinib alleviated GM-CSF-induced joint inflammation between days 6 and 7 (Suppl-3D). Overall, we showed that tofacitinib inhibits GM-CSF-induced inflammatory and glycolytic profiles in both the experimental model and RA MΦs.

## Discussion

GM-CSFRα/β enrichment in RA synovial lining and sublining CD68⁺MΦs correlates with disease activity, highlighting GM-CSF responsiveness. GM-CSF reprogramming of RA blood and synovial tissue MΦs revealed overlapping and distinct features. RA blood and synovial MΦs exposed to GM-CSF exhibit an IL1β^+^S100A^+^HIF1^+^IL10^lo^NFIL3/6^lo^ profile, along with a metabolic shift favoring glycolysis over oxidative phosphorylation, resulting in hyperglycolysis (glycoATP^high^/mitoATP^low^) and mitochondrial fragmentation. While anti-TNFi and anti-IL6R Abs had minimal impact on the GM-MΦ signature, HK2 inhibition restored GM-CSF-suppressed TCA enzymes. Intriguingly, Tofacitinib disruption of GM-CSFRα/STAT5 signaling redirects IL1β^hi^S100A^hi^HIF1^hi^IL10^lo^NFIL3/6^lo^ MΦs to a regulatory counterpart, in concert with rescuing oxidative stress and mitochondrial fragmentation. Extending these findings, tofacitinib attenuated GM-CSF-induced arthritis by constraining myeloid inflammatory and mitochondrial landscapes in the joints via IL1β, pSTAT5, HIF1α, and DRP1 reduction (Fig. 8J).

### Effect of standard-of-care therapy on GM-MΦs

We and others showed that the overexpression of GM-CSF in RA synovial fluid is primarily due to the activation of lymphocytes in response to IL17 (Th17 cells) [36], IL7/STAT5 (ThGM) [9], or RA joint-specific Th1 cells [37]. Yet, RA MΦs overexpress GM-CSF and GM-CSFRα when stimulated by LPS. ScRNAseq and histological analysis uncovered that GM-CSFRα/β are enriched in the RA synovial M1(IL1β^+^)-MΦ and M4(IFN^+^)-MΦ subpopulations [33], where they colocalize with STAT5, HBEGF, S100, and HIF1α, forming a pro-inflammatory and glycolytic hub. Corroborating this notion, STAT5 deactivation via tofacitinib realigns GM-CSF-reprogrammed RA IL1β^+^HBEGF^+^S100A^+^ cells into regulatory MERTK^+^NFIL3/6^+^ MΦs, which exhibit oxidative repair capabilities.

Previous studies postulated that DMARD therapy could reduce joint T-cell recruitment and their ability to secrete GM-CSF [38, 39] and activate JAK/STAT [40]. Contrary to these findings, methotrexate (MTX) promotes rather than reduces GM-CSF transcription in IL1β-stimulated RA FLS via NF-κB and IRAK4 signaling [41]. Nonetheless, in a Phase IIB study, MTX plus GM-CSFRα Ab (Mavrilimumab, 150 mg) showed greater ACR20 compared to MTX alone [42, 43], suggesting that DMARDs may function independently of GM-CSF-mediated pathology. Consistently, our data indicate that DMARDs exert little to no effect on GM-CSF and GM-CSFRα/β expression in RA synovial tissues.

Recent studies have illustrated that while TNF is implicated in GM-CSF-mediated arthritic pain in the early phase, it does not influence late disease [44]. The crosstalk between TNF and GM-CSF has been documented in preclinical arthritis models [44]. Nevertheless, serum GM-CSF levels remain elevated in anti-TNFi-responsive RA patients [45], indicating that GM-CSF could be modulated by other mechanisms, including Th17 or ThGM cells [9, 36]. Interestingly, differential efficiency was observed for GM-CSF Ab (Otilimab) compared to GM-CSFRα Ab (Mavrilimumab) in DMARD or anti-TNFi non-responders (EARTH EXPLORER 2, a Phase IIB trial) [46]. Consistent with our findings, tofacitinib demonstrated superior efficacy and safety compared to the GM-CSF antibody, Otilimab, regardless of GM-CSF dependency in RA patients [47].

Other biologics, such as anti-IL6R Ab, may indirectly disrupt GM-CSF function through JAK/STAT signaling [48]. Alternatively, GM-CSF and IL6 originate from Th17 cells [49–51]; hence, GM-CSF blockade may also cross-regulate IL6 function. In line with our findings, IL6 emerges as a hallmark of GM-CSF-MΦ reprogramming in RA blood, synovial tissue, and preclinical arthritis, with tofacitinib significantly reducing its levels across all models. In contrast to tofacitinib, anti-IL6R Ab and anti-TNFi therapies had no effect on synovial GM-CSFR expression and only a modest impact on the GM-MΦ signature profile.

### GM-MΦs imprinting in RA

In GM-MΦs, ERK, p38, and STAT5 are activated without influencing NF-κB, JAK1/3, or STAT1/3 signaling. Distinctly, dendritic cell differentiation by GM-CSF is dependent on NF-κB proteins RelA and p50 [52, 53]. RA GM-MΦs display expansion of a wide range of monokines, CCL1, CCL3, CCL17, CCL23, CCL24, and CXCL5, in concert with IRF7 transcription. Strikingly, CCL17 and CCL24 displayed the most prominent profiles in GM-MΦs. At the same time, reduced CCL5 and CXCL10 transcription in RA GM-MΦs coincided with IRF1/3/4/8 downregulation. Conversely, others have shown that GM-CSF-reprogrammed MΦs were associated with IRF4-induced CCL17 in zymosan-induced arthritis [44]. Nonetheless, recent clinical trials targeting CCL17 have demonstrated only modest benefits [54, 55], highlighting the complexity of this pathway and reinforcing the rationale for focusing on GM-CSF as a more central therapeutic target.

Transformative studies have pathotyped various MΦ subpopulations in RA synovium during active disease and remission [33–35]. Recent reports have shown that RA synovial CD40-enriched IL1β^+^CCL20^+^ and SPP1^+^MT2A^+^ MΦ subtypes are critical for stromal cell responses [56]. Uniquely, RA blood and synovial tissue reconfigured by GM-CSF exhibit an oxidatively stressed IL1β^+^S100A^+^HIF1^+^IL10^lo^NFIL3/6^lo^ signature accompanied by STAT5 signaling. GM-MΦs promote hyperglycolysis (↑HK2, ↑LDH, ↑glycoATP) over oxidative phosphorylation (↓OGDH, ↓SDH, ↑HIF1α, ↓mitoATP), advancing citrate, succinate, and lactate accumulation and mitochondrial dynamic changes (↑DRP1, ↓MFN2). To repair the inflammatory and metabolic dysfunction, GM-MΦs and GM-CSF-activated RA synovial tissues were treated with HK2i, or tofacitinib. HK2i restored the levels of regulatory factors (MERTK, MARCO, NUPR1, IL10, NFIL3/6, BLIMP) and TCA enzymes (IDH, OGDH, SDHA) in GM-MΦs; nevertheless, it failed to rescue inflammatory mediators (except CCL1/2) or correct the excessive mitochondrial fragmentation. Likewise, HK2i partially reversed GM-CSF-reprogrammed RA synovial tissues by upregulating NFIL6 and suppressing IL1β, CCL2, and S100A8, while leaving GM-CSFR, and other inflammatory signatures (IL6 and S100A9) unaffected. Through ARG2, IL10 impairs succinate, and IL1β in LPS-differentiated bone marrow (BM) MΦs [57]. Consistently, IL10 preserves TCA function in LPS-reprogrammed BM-MΦs by blunting nitric oxide (NO) production [58]. Our data suggest that the heightened glycoATP in GM-MΦs remedied by HK2i may be connected to IL10 effector genes (NFIL3/6, BLIMP) rebuilding the regulatory (MERTK, MARCO, NUPR1) and oxidative genes (IDH, OGDH, SDHA) suppressed in GM-CSF-reprogramming in RA blood and/or synovial tissues. Conversely, inflammatory, oxidative, and mitochondrial dynamics altered by GM-MΦs remained unchanged in response to Complex li (except CCL2).

### Impact of Tofacitinib on GM-MΦs

Similar to Rinvoq, tofacitinib targets a broad spectrum of JAK/STAT pathways and promotes a shift of GM-MΦs toward a regulatory phenotype. Specifically, disruption of STAT5 signaling by tofacitinib downregulates synovial GM-CSFR expression and suppresses inflammatory and glycolytic mediators in both GM-MΦs and RA synovial tissue. Moreover, tofacitinib redirects cellular energy production from glycoATP to mitoATP, rescues oxidative metabolic enzymes (IDH, OGDH, SDH), restores IL10-associated regulatory programs (NFIL3/6), and normalizes mitochondrial dynamics in GM-MΦs and RA synoviocytes reprogrammed by GM-CSF. Nonetheless, tofacitinib was inconsequential on p38 signaling in GM-MΦs. Extending our findings, patients receiving tofacitinib displayed lower STAT1/3/4 and STAT5 signaling in RA PBMCs, which was associated with decreased DAS28 response and lower IL6, DNER, and CCL11 [59, 60]. RA FLS, unlike RA MΦs, do not respond to GM-CSF or to conditioned media from GM-MΦs.

In RA CD14^+^CXCR3^+^ myeloid cells, tofacitinib, but not anti-TNFi, was involved with cell migration due to the influence of JAK/STAT signaling on chemokine receptors [61]. These investigators also demonstrate that remodeling of monocytes into glycolytic MΦs in response to synovial explant-conditioned media occurs through STAT signaling [62]. RA GM-CSFRα/β^+^ MΦs signal through STAT5, which rewires a distinct subset of glycolytic myeloid cells that coexpress HBEGF, S100A, and HIF1α.

While STAT3 blockade was inconsequential on LPS/IFNγ-induced ECAR (glycoATP), it negated the LPS/IFNγ-mediated suppression of OCR (mitoATP) [63]. HIF1α suppression by STAT3i in LPS/IFNγ-differentiated MΦs [64] may be responsible for the interception of inflammatory and metabolic traits. Differentially, in GM-MΦs, escalated glycoATP over mitoATP was counteracted by tofacitinib, coinciding with a reduction of lactate, ROS, and HIF1α. Correspondingly, tofacitinib suppressed joint GLUT1 and HK2, while reducing F4/80⁺HBEGF⁺HIF1α⁺MΦs in GM-CSF-induced arthritic mice, contributing to decreased IL1β, CCL2, and CCL5 transcription. Distinct from GM-MΦs and RA synoviocytes, tofacitinib did not restore IL10 or oxidative enzymes (IDH, OGDH, SDHA) in GM-CSF-induced arthritis. However, tofacitinib therapy mitigated GM-CSF-induced joint swelling, inflammatory, glycolytic, and oxidative stress factors, including HIF1α. Tofacitinib corrected the elevated DRP1-to-MFN2 ratio and oxidative stress imbalance in GM-MΦs from RA specimens and a preclinical model. Our findings show that preventative and therapeutic tofacitinib treatment alleviates GM-CSF-induced joint inflammation.

Overall, our results indicate that GM-CSFRα/β⁺ MΦs in RA synovial tissue respond to tofacitinib by promoting regulatory and oxidative repair genes, while suppressing inflammatory and glycolytic pathways via STAT5 deactivation.

## Materials and methods

### Study design

This study aimed to uncover the effect of GM-CSF on RA MΦ immunometabolism and find strategies to repair mitochondrial dysfunction. To ensure a robust and unbiased experimental design, samples were obtained from RA patients or mice of both genders. Mice used within the same experimental group were aged 8–10 weeks and sex matched. Rigor and reproducibility were maintained through well-powered studies and multiple distinct approaches to confirm the results. Power analysis was performed by setting 90% power at the statistical significance level α = 0.05 based on the effect sizes of our previous studies [2, 65].

### Human peripheral blood, synovial fluid, and synovial tissue from RA patients

RA and OA synovial fluids and RA peripheral blood and synovial tissue specimens were obtained in accordance with the University of Illinois Chicago Institutional Ethics Review Board-approved protocol (IRB protocol# 2022-0396). RA patients were diagnosed according to the 1987 revised criteria of ACR [66]. All patients gave written informed consent before blood or synovial fluid was drawn or synovial tissue was extracted. All adults diagnosed with RA were included in this study, regardless of sex, age, treatment regimen, or demographic information, which were not part of our experimental design.

### Cells and stimulation/inhibition

Peripheral blood mononuclear cells (PBMCs) were isolated by density gradient centrifugation using Ficoll-Paque PREMIUM (GE Healthcare) from the buffy coat and subsequently used for further analysis. PBMCs were cultured in 20% fetal bovine serum/RPMI for 2–3 days to obtain in vitro differentiated naive MΦs. To differentiate RA monocytes into MΦs, 3–4 × 10^6^ cells/well were cultured for 2 days using RPMI (Thermo Fisher) supplemented with 10% FBS, 100 U/ml of penicillin, 100 μg/ml streptomycin, and 2 mM glutamine. For inhibition, MΦs were serum starved o/n, in the presence of HK2i (2-deoxy-D-glucose, 5 mM, Sigma-Aldrich), Complex Ii (IACS-010759, 100 nM, ChemieTek), tofacitinib (10 μM, APExBio), Anti-IL6R Ab (Tocilizumab, 10 μg/ml), Anti-TNFi (10 μg/ml, R&D System), Rinvoq (10 μM, AmBeed), or Stat5i (1 μM, AmBeed), then they were either untreated or treated with GM-CSF (100 ng/ml, Biolegend) for 5 min to 24 h. Cells were subsequently harvested in TRIzol reagent (Life Technologies) or RIPA buffer (Cell Signaling Technology) for mRNA quantification and western blot analysis, respectively; conditioned media was collected for ELISA or colorimetric assay.

### RA explant

RA synovial tissues (25 mg) were cut into small pieces of approximately 2–3 mm³ to ensure proper access to stimuli and were starved o/n in RPMI containing 0% FBS, with or without tofacitinib and HK2i. Next, the synovial tissues were stimulated with GM-CSF for 6 h. All reagents were used at twofold higher concentrations in RA explants compared to monocyte-differentiated MΦs in culture. Thereafter, tissues were harvested for mRNA extraction and transcript-level analysis.

### Bulk and single-cell RNA sequencing

RA PBMCs were either untreated or treated with 100 ng/ml of GM-CSF for 12 h. Total mRNA was isolated using TRIzol reagent and sent to Admera Health (South Plainfield, NJ) for quality control, library preparation, sequencing by synthesis, and bioinformatics analysis. RNA with an RNA integrity number (RIN) score >7 was used. Library construction was performed according to the NEBNext® Ultra™ II Directional RNA Library Prep Kit for Illumina®. The final library quantity was assessed by Qubit 2.0 (Thermo Fisher), and its quality was evaluated by TapeStation (Agilent Technologies). Paired-end sequencing was performed using the Illumina NovaSeq 6000, yielding approximately 60 million paired-end reads per sample. Next, sequence reads were mapped to the human genome (GRCh38) reference using the STAR aligner. The raw reads were generated using HT-Seq Count (2.0.5). Normalization calculated the Fragments per kilobase per million mapped reads (FPKM) and transcripts per kilobase million (TPM) values for each gene to correct for sequencing depth and length using StringTie. Differential expression analysis was performed using the DESeq2 R package (version 1.18.1) and Benjamini-Hochberg-adjusted *p*-values to control the false discovery rate at *p* < 0.05. The differentially expressed genes are defined by a cut-off adjusted *p*-value of 0.05 and an absolute (Log2FC) value of 1.0. The enrichment analysis was conducted using Gene Ontology (GO) and KEGG pathway maps with ClusterProfiler 4.0 software [67, 68]. Data is available publicly through the NCBI GEO database under Accession#GSE288373.

The RNAseq dataset GSE130567 was deposited by Kang et al. [69] was accessed using the web interface https://www.ncbi.nlm.nih.gov/geo/query/acc.cgi?acc=GSE130567 to evaluate the expression of GMCSF-Rα, GMCSF-Rβ, and GMCSF in M-CSF differentiated CD14^+^ monocytes treated with LPS, IFNγ, or primed with IFNγ then activated with LPS compared with controls. The web interface at https://peac.hpc.qmul.ac.uk/, developed by Lewis et al. [4], was used to assess the expression levels of GM-CSF, GM-CSFRα, and GM-CSFRβ in synovial tissues from early rheumatoid arthritis (RA) patients involved in the PEAC study.

The RNA-seq dataset GSE198520 https://www.ncbi.nlm.nih.gov/geo/geo2r/?acc=GSE198520 [70] was used to evaluate the expression of GM-CSF, GM-CSFRα, and GM-CSFRβ in RA synovium biopsied from 35 RA patients 12 weeks after anti-TNFi (Etanercept).

The web interface https://r4ra.hpc.qmul.ac.uk/ [71, 72] was used to evaluate the expression of GM-CSF, GM-CSFRα, and GM-CSFRβ in RA synovial tissues treated with Tocilizumab (anti-IL6R Ab).

The single-cell RNA sequencing data from Zhang et al. [33] were accessed from the Broad Institute Single Cell portal at the following URL: https://singlecell.broadinstitute.org/single_cell/study/SCP279/amp-phase-1. This cohort consisted of RA patients who were older than 18 years and had at least one inflamed joint. The RNAseq dataset identifying differentially expressed genes during flare was accessed from Supplementary Appendix 2, published by Orange et al. [5].

### Quantitative RT‑PCR

RNA was isolated using TRIzol reagent (Life Technologies) following the manufacturer’s instructions. The isolated RNA was then reverse transcribed into cDNA using the High-Capacity cDNA Reverse Transcription Kit (Applied Biosystems). qRT-PCR analysis was conducted using TaqMan Gene Expression Master Mix (Applied Biosystems). Predesigned IDT primers were utilized, as listed in Tables S1 (human primers) and S2 (murine primers). The data are presented as fold change (S1^−ΔΔCt^), normalized to the housekeeping gene β-actin, and compared to the untreated control. Data were acquired using the QuantStudio5 (Applied Biosystems) qRT-PCR machine.

### ELISA and Metabolite evaluation

Human IL1β, IL6, CCL2, and IL10 protein levels were quantified by ELISA, following the manufacturer’s instructions (R&D Systems) in culture supernatants of RA-MΦs. According to the manufacturer’s instructions, lactate, citrate, and succinate were measured in conditioned media using colorimetric assay kits for lactate (MAK329, Sigma), succinate (MAK335, Sigma), and citrate (MAK333, Sigma).

### Western blot analysis

Samples were lysed in RIPA buffer (Cell Signaling Technology) supplemented with protease and phosphatase inhibitors (Roche), and protein concentration was determined using the Pierce BCA Protein Assay Kit (Thermo Fisher) according to the manufacturer’s instructions. Lysates were resolved on 10% polyacrylamide gels and transferred using the Trans-Blot Turbo Transfer System (Bio-Rad). Blots were subsequently probed for pERK, NF-κBp65, NF-κBp50, p-p38, pSTAT1/3/5, JAK1, JAK3, GLUT1, HK2, MFN2, and DRP1 (Cell Signaling), actin (Santa Cruz), and anti-rabbit IgG HRP-linked or anti-mouse IgG HRP-linked secondary antibodies (Cell Signaling, Table S3). Detection was performed using the iBright 1500 imaging system (Invitrogen by Thermo Fisher). Signal intensities were normalized to β-actin as a loading control and subsequently to the baseline (0 h) to determine relative fold changes.

### Seahorse ATP rate assay

GlycoATP and mitoATP were measured using the Seahorse XF ATP rate assay kit (Agilent Technologies) per the manufacturer’s instructions. PBMCs (0.64 × 10^6^ cells/well) were untreated or treated with GM-CSF (1 μg/ml) with or without Tofacitinib (100 μM), Rinvoq (100 μM), or Stat5i (100 μM) o/n. ATP production was calculated using the following equation: ATP production = (Last rate measurement before Oligomycin injection) – (Minimum rate measurement after Oligomycin injection). GlycoATP production and mitoATP production were measured using the Seahorse XF ATP Rate Test kit (Agilent Technologies), according to the manufacturer’s instructions.

### Flow cytometry

Monocytes were enriched from RA PBMCs by negative selection using the EasySep™ Human Monocyte Isolation Kit (StemCell Technologies) according to the manufacturer’s protocol. Treated cells were stained with Zombie Violet (BioLegend) for live/dead discrimination and with anti-CD14-FITC and anti-CD86-APC antibodies (BioLegend) for 30 min at 4 °C in the dark. After staining, cells were washed, resuspended in staining buffer, and acquired on a Gallios 10/3 flow cytometer (Beckman Coulter) at the University of Illinois Chicago Flow Cytometry Core Facility. Data were analyzed using Kaluza software (Beckman Coulter) [2].

### ROS quantification

To evaluate ROS, monocytes were plated in a black-walled, clear-bottom 96-well plate at a density of 300,000 cells/well. The cells underwent serum starvation o/n in the presence of Tofacitinib, followed by GM-CSF stimulation. ROS levels were measured using the cell-permeable reagent 2′,7′-dichlorofluorescein diacetate (DCFDA, Abcam) according to the manufacturer’s protocol. The generation of intracellular ROS was evaluated through microscopy, and subsequent data analysis was conducted using ImageJ.

### Histological studies

Formalin-fixed, paraffin-embedded RA synovial tissue was sectioned and subjected to immunostaining for the co-localization of CD68, GM-CSFR, pSTAT5, HBEGF, S100A12, MFN2, DRP1, and HIF1α (Table S4). Subsequently, secondary labeling was done using anti-rabbit and anti-mouse antibodies to visualize the staining.

Formalin-fixed mouse ankles were decalcified and paraffin-embedded. Slides were deparaffinized in xylene, and antigen retrieval was achieved as previously described [65, 73]. Mouse ankle sections were stained for H&E, F4/80, HBEGF, GLUT1, HK2, HIF1α, pSTAT5, MFN2, DRP1, and TOM20 (Table S4) and were scored on a scale of 0-5 in a blinded manner [65, 74].

RA monocytes or differentiated MΦs were cultured on glass coverslips and treated with 100 ng/ml GM-CSF ± 10 µM Tofacitinib for 6 h. Thereafter, cells were fixed using 3.7% paraformaldehyde for 10 min, washed, and permeabilized with 0.1% saponin for 1 h. Cells were stained with pSTAT5 and CD14 for 1 h (Table S4). Fluorescence secondary anti-mouse antibodies and DAPI were used to visualize staining.

To assess mitochondrial dynamics, RA MΦs were plated at a density of 2 × 10^6^ cells/well on a coverslip in a 24-well plate. After 48 h, cells were serum-starved overnight and either untreated or treated with HK2i (5 mM) or tofacitinib (10 μM), before being stimulated with GM-CSF (100 ng/ml) for 6 h. Subsequently, cells were stained with TOM20, MFN2, or DRP1 (Table S4). Cells were imaged with 20 z-stacks per image at 0.5 μm per z-stack for 10 μm total, as described previously [75].Deconvolved images were analyzed using Imaris x64 (v7.6.4; Bitplane). The Spots function in Imaris was used to quantify the signal of interest (TOM20) using an estimated spot diameter of 0.75 µm.

### RA MΦs-FLS crosstalk studies

RA MΦs were cultured and either left untreated (Ctrl) or stimulated with LPS (100 ng/ml) or GM-CSF (100 ng/ml) for 24 h. Thereafter, conditioned media (CM) was collected, centrifuged to remove cell debris, and applied to cultured fibroblast-like synoviocytes (FLS) for 6 h. Following stimulation, FLS were harvested, followed by RNA extraction and transcript level analysis of IL6 and CCL2 by qRT-PCR.

### Animal studies

All animal studies were approved by the University of Illinois Chicago Animal Care and Use Committee 25-014. Eight to ten-week-old WT C57BL/6 mice were used for all experiments. Mice received i.p. injections of tofacitinib (10 mg/kg BW) daily from day −2 through day 10. On day 0, the mice were intra-articularly (i.a.) injected with adenovirus (Ad)-Ctrl and Ad-GM-CSF at a dose of 3 × 10^10^ viral particles (vp) per ankle. On day 7, the animals received a booster of Ad-Ctrl or Ad-GM-CSF (1.5 × 10^10^vp). Ankle circumference was measured from day 1 to 10 using a digital caliper, and mice were sacrificed on day 10. Ankle circumference was determined by measuring two perpendicular diameters: the laterolateral (ML) and anteroposterior (AP) diameters. Circumference was then calculated using the following formula: circumference=2π x (sqrt (a^2^ + b/2)), where a and b represent the diameters [76].

For therapeutic treatment, mice were injected i.a. with Ad-Ctrl or Ad-GM-CSF (3 × 10¹⁰ vp per ankle) on day 0. Beginning on day 2 post-onset, mice received daily i.p. injections of tofacitinib (10 mg/kg BW) through day 7. Ankle circumference was measured daily to monitor disease progression.

### Statistical analysis

Data are presented as mean ± SEM. All statistical tests were two-sided, with *p* < 0.05 considered statistically significant. Comparisons between two groups were performed using the nonparametric Mann–Whitney test, while comparisons among more than two groups were performed using one-way ANOVA followed by Tukey’s multiple comparisons test, or the Kruskal–Wallis test followed by Dunn’s multiple comparisons test for nonparametric data. For analyses involving multiple groups with two factors, two-way ANOVA was applied, and *p*-values from multiple comparisons were adjusted using Tukey’s method [77]. The statistical tests applied to individual datasets are specified in the corresponding figure legends. All analyses were performed using GraphPad Prism version 10 in consultation with Dr. Xia.

## Supplementary information

Table 1-5

Supplementary data&unprocessed images

Supplementary figure captions

## Data Availability

All findings are exhibited in the paper, and the material and data are available for transparency. RNAseq data is available publicly through the NCBI GEO database under Accession#GSE288373.
