# Supplementary material for: Tofacitinib repairs inflammation and mitochondrial dysregulation in GM-CSF-reprogrammed RA macrophages
Source: Cell Mol Immunol. 2026 Mar 4;23(4):417–31. doi: 10.1038/s41423-026-01395-x (PMC13035809; doi:10.1038/s41423-026-01395-x)
Supplement: Supplementary file 1 — Table 1-5 [file 41423_2026_1395_MOESM1_ESM.docx]

**TABLE S1: Predesigned IDT primers and TaqMan gene expression assays used to quantify mRNA expression of human samples.**

| *Gene Name* | *Assay ID* | *Company* | *Ref Seq #* | *Assay Configuration* |
| --- | --- | --- | --- | --- |
| *ACTB* | Hs.PT.56a.3404650.g | IDT | NM_001101 | Std, FAM/ZEN/IBFQ, P:P 2 |
| *IL1β* | Hs.PT.58.40913627 | IDT | NM_000575 | Std, FAM/ZEN/IBFQ, P:P 2 |
| *IL6* | Hs.PT.58.40226675 | IDT | NM_000600 | Std, FAM/ZEN/IBFQ, P:P 2 |
| *IL10* | Hs.PT.58.2807216 | IDT | NM_000572 | Std, FAM/ZEN/IBFQ, P:P 2 |
| *cMAF* | Hs.PT.58.872936 |  | NM_001031804(1) | Std, FAM/ZEN/IBFQ, P:P 2 |
| *BLIMP* | Hs.PT.56a.39313533.g | IDT | NM_182907(2) | Std, FAM/ZEN/IBFQ, P:P 2 |
| *NFIL3* | Hs.PT.58.4948721 | IDT | NM_005384(1) | Std, FAM/ZEN/IBFQ, P:P 2 |
| *NFIL6 (CEBPB)* | Hs.PT.58.27185099.g | IDT | NM_005194(1) | Std, FAM/ZEN/IBFQ, P:P 2 |
| *CCL1* | Hs.PT.58.40788777 | IDT | NM_002981(1) | Std, FAM/ZEN/IBFQ, P:P 2 |
| *CCL2* | Hs.PT.58.45467977 | IDT | NM_002982 | Std, FAM/ZEN/IBFQ, P:P 2 |
| *CCL3* | ACC AGT TCT CTG CAT CAC TTG  GCT GCT CGT CTC AAA GTA GTC | IDT | /56-FAM/TC CCGGCA G/ZEN/A TTC CAC AGA ATT TCA/3IABkFQ | Std, FAM/ZEN/IBFQ, P:P 2 |
| *CCL5* | Hs.PT.58.1724551 | IDT | NM_002985 | Std, FAM/ZEN/IBFQ, P:P 2 |
| *CCL17* | Hs.PT.58.159549 | IDT | NM_002987(1) | Std, FAM/ZEN/IBFQ, P:P 2 |
| *CCL23* | Hs.PT.58.39087443.g | IDT | NM_005064(2) | Std, FAM/ZEN/IBFQ, P:P 2 |
| *CCL24* | Hs.PT.58.395772682.g | IDT | NM_002991(1) | Std, FAM/ZEN/IBFQ, P:P 2 |
| *CXCL5* | Hs.PT.58.41058007.g | IDT | NM_002994 | Std, FAM/ZEN/IBFQ, P:P 2 |
| *CXCL10* | Hs.PT.58.3790956.g | IDT | NM_001565(1) | Std, FAM/ZEN/IBFQ, P:P 2 |
| *IRF1* | Hs.PT.58.26847423 | IDT | NM_002198 | Std, FAM/ZEN/IBFQ, P:P 2 |
| *IRF3* | Hs.PT.58.19791480 | IDT | NM_001197124 | Std, FAM/ZEN/IBFQ, P:P 2 |
| *IRF4* | Hs.PT.58.2073651 | IDT | NM_001195286 | Std, FAM/ZEN/IBFQ, P:P 2 |
| *IRF5* | Hs.PT.58.20692129 | IDT | NM_001242452 | Std, FAM/ZEN/IBFQ, P:P 2 |
| *IRF7* | Hs.PT.58.24613215.g | IDT | NM_001572 | Std, FAM/ZEN/IBFQ, P:P 2 |
| *IRF8* | Hs.PT.58.455867 | IDT | NM_002163 | Std, FAM/ZEN/IBFQ, P:P 2 |
| *HBEGF* | Hs.PT.58.20429993 | IDT | NM_001945(1) | Std, FAM/ZEN/IBFQ, P:P 2 |
| *S100A8* | Hs.PT.58.19654111.gs | IDT | NM_002964(1) | Std, FAM/ZEN/IBFQ, P:P 2 |
| *S100A9* | Hs.PT.58.41787562 | IDT | NM_002965(1) | Std, FAM/ZEN/IBFQ, P:P 2 |
| *S10012* | Hs.PT.58.39942209 | IDT | NM_005621(1) | Std, FAM/ZEN/IBFQ, P:P 2 |
| *MERTK* | Hs.PT.58.2640315 | IDT | NM_006343(1) | Std, FAM/ZEN/IBFQ, P:P 2 |
| *MARCO* | Hs.PT.58.3036594 | IDT | NM_006770(1) | Std, FAM/ZEN/IBFQ, P:P 2 |
| *NUPR1* | Hs.PT.58.19949540 | IDT | NM_00104248 | Std, FAM/ZEN/IBFQ, P:P 2 |
| *ADORA3* | Hs.PT.56a.223977 | IDT | NM_00108197 | Std, FAM/ZEN/IBFQ, P:P 2 |
| *SPP1* | Hs.PT.58.19252426 | IDT | NM_000582(5) | Std, FAM/ZEN/IBFQ, P:P 2 |
| *ACO2* | Hs.PT.56a.45342094 | IDT | NM_001098(1) | Std, FAM/ZEN/IBFQ, P:P 2 |
| *CS* | Hs.PT.58.40916376 | IDT | NM_004077(1) | Std, FAM/ZEN/IBFQ, P:P 2 |
| *IDH* | Hs.PT.58.2561570 | IDT | NM_005896(1) | Std, FAM/ZEN/IBFQ, P:P 2 |
| *OGDH* | Hs.PT.58.25912455 | IDT | NM_001165036(2) | Std, FAM/ZEN/IBFQ, P:P 2 |
| *SDHA* | Hs.PT.58.410177719 | IDT | NM_004168(1) | Std, FAM/ZEN/IBFQ, P:P 2 |
| *FH* | Hs.PT.58.40261049 | IDT | NM_000143(1) | Std, FAM/ZEN/IBFQ, P:P 2 |
| *MDH2* | Hs.PT.58.38847350 | IDT | NM_005918(1) | Std, FAM/ZEN/IBFQ, P:P 2 |
| *HIF1α* | Hs.PT.58.534274 | IDT | NM_001243084 | Std, FAM/ZEN/IBFQ, P:P 2 |
| *DRP1 (DNM1L)* | Hs.PT.58.5037880 | IDT | NM_005690(3) | Std, FAM/ZEN/IBFQ, P:P 2 |
| *MFN2* | Hs.PT.58.14928618 | IDT | NM_001127660(2) | Std, FAM/ZEN/IBFQ, P:P 2 |
| *GLUT1* | Hs.PT.58.25872862 | IDT | NM_006516 | Std, FAM/ZEN/IBFQ, P:P 2 |
| *HK2* | Hs.PT.58.4863675 | IDT | NM_000189 | Std, FAM/ZEN/IBFQ, P:P 2 |
| *LDHA* | Hs.PT.58.22929122 | IDT | NM_001165416 | Std, FAM/ZEN/IBFQ, P:P 2 |

TABLE S2: Predesigned IDT primers and TaqMan gene expression assays used to quantify mRNA expression of murine samples.

| *Gene name* | *Assay ID* | *Company* | *Ref Seq #* | *Assay Configuration* |
| --- | --- | --- | --- | --- |
| *GAPDH* | Mm03302249 |  | PN4351368 | GM10481;G+FAM |
| *IL1β* | Mm.PT.58.41616450 | IDT | NM_008361(1) | Std, &#160; FAM/ZEN/3' Iowa Black FQ, P:P 2 |
| *IL6* | Mm.PT.58.10005566 | IDT | NM_031168(1) | Std, &#160; FAM/ZEN/3' Iowa Black FQ, P:P 2 |
| *CCL2* | Mm.PT.58.42151692 | IDT | NM_011333(1) | Std, &#160; FAM/ZEN/3' Iowa Black FQ, P:P 2 |
| *CCL5* | Mm01302427 |  |  |  |
| *TGFβ* | Mm.PT.58.11254750 | IDT | NM_011577(1) | Std, &#160; FAM/ZEN/3' Iowa Black FQ, P:P 2 |
| *IL10* | Mm.PT.58.13531087 | IDT | NM_010548(1) | Std, &#160; FAM/ZEN/3' Iowa Black FQ, P:P 2 |
| *IDH* | Mm.PT.58.5996441 | IDT | NM_001111320(2) | Std, &#160; FAM/ZEN/3' Iowa Black FQ, P:P 2 |
| *OGDH* | Mm.PT.58.7792999 | IDT | NM_001252282(3) | Std, &#160; FAM/ZEN/3' Iowa Black FQ, P:P 2 |
| *SDHA* | Mm.PT.56a.12170577 | IDT | NM_023281(1) | Std, &#160; FAM/ZEN/3' Iowa Black FQ, P:P 2 |

**TABLE S3: Complete list of antibodies used for protein detection by western blot analysis.**

| *Antibody* | *Company* | *Catalog #* | *Host* | *Species reactivity* |
| --- | --- | --- | --- | --- |
| *Actin* | SantaCruz | Sc-47778 | mouse | human, Mouse |
| *Phospho-p44/42 MAPK (Erk1/2) (Thr202/Tyr204)* | Cell Signaling Technology | #9101 | rabbit | human, Mouse |
| *RELA/NFκB p65 (F-6)* | SantaCruz | Sc-8008 | mouse | human, mouse |
| *NFκB p50* | SantaCruz | sc-8414 | mouse | human, mouse |
| *P-p38 MAPK (Thr180/Tyr182)* | Cell Signaling Technology | #9211 | rabbit | human, mouse |
| *pStat1 (Tyr701) (D4A7)* | Cell Signaling Technology | #7649S | rabbit | human, mouse |
| *pStat3 (Tyr705)* | Cell Signaling Technology | #9131S | rabbit | human, mouse |
| *pStat5 (Tyr694) (C11C5)* | Cell Signaling Technology | #9359S | rabbit | human, mouse |
| *pJak1 (Tyr1034/1035)* | Cell Signaling Technology | #3331S | rabbit | human, mouse |
| *pJak3 (Tyr980/981)* | Cell Signaling Technology | #5031S | rabbit | human, mouse |
| *Mitofusin-2 (D2D10) (MFN2)* | Cell Signaling Technology | #9482 | rabbit | human, mouse |
| *DRP1 (D8H5)* | Cell Signaling Technology | #5391 | rabbit | human, mouse |
| *Glut1 (D3J3A)* | Cell Signaling Technology | #12939 | rabbit | human, mouse |
| *Hexokinase II (C64G5) (HK2)* | Cell Signaling Technology | #2867 | rabbit | human, mouse |
| *Anti-Rabbit IgG HRP Conjugate* | Promega | W4011 | NA | NA |
| *Anti-Mouse IgG HRP Conjugate* | Promega | W4021 | NA | NA |

**TABLE S4**: **Primary Antibodies used for protein detection by immunohistochemistry.**

| Antibody | Company | Catalog # | Host | Species Reactivity | *Conc.* |
| --- | --- | --- | --- | --- | --- |
| *CD68* | Dako | M0879 | mouse | human | 1:100 |
| *GM-CSFR* | SantaCruz | sc-456 | mouse | mouse, rat and human | 1:50 |
| *HBEGF* | SantaCruz | sc-365182 | mouse | mouse, rat and human | 1:50 |
| *S100A12* | SantaCruz | sc-101347 | mouse | human | 1:50 |
| *GLUT1* | Cell Signal | #12939S | rabbit | mouse, rat and human | 1:100 |
| *HK2* | SantaCruz | sc-374091 | rabbit | human | 1:50 |
| *HIF1α* | SantaCruz | sc-53546 | mouse | mouse, rat and human | 1:50 |
| *TOM20* | SantaCruz | sc-117764 | mouse | human | 1:50 |
| *MFN2* | Cell Signal | #9482S | rabbit | Human, Mouse, Rat, Hamster, Monkey | 1:200 |
| *DRP1* | SantaCruz | sc-271583 | mouse | mouse, rat and human | 1:50 |
| *pSTAT5* | Cell Signal | #9339 | rabbit | Human, Mouse | 1:300 |
| *F4/80* | Genetex | GTX26640 | rat | Human, Mouse, Rat | 1:100 |
| *CD14* | Proteintech | #60253-1-Ig | mouse | human | 1:100 |
| *VECTASHIELD Vibrance Antifade Mounting Medium with DAPI* | Vector Laboratories | #H-1800 | N/A | N/A |  |
| *Alexa Fluor 488 AffiniPure F(ab’)_2_ Fragment (green)* | Jackson ImmunoResearch | #715-546-150 | donkey | mouse |  |
| *Alexa Fluor 594 AffiniPure F(ab’)_2_ Fragment (red)* | Jackson ImmunoResearch | #711-586-152 | donkey | mouse |  |

**TABLE S5: Complete list of reagents and resources.**

| Reagent | Source | Identifier |
| --- | --- | --- |
| *PBS* | Corning | #21-040-CM |
| *RPMI* | Corning | #10-040-CV |
| *Ficoll paque premium* | Millipore Sigma | #17544203 |
| *Recombinant human GM-CSF (carrier free)* | Biolegend | #572904 |
| *2-Deoxy-D-glucose* | Sigma | D8375 |
| *IACS-010759* | Chemietek | CT-IACS107 |
| *Tofacitinib* | Apexbio | CP-690550 |
| *Anti-IL6R (Tocilizumab)* | Genentech | ***-*** |
| *Human TNF-alpha Antibody* | R&D System | MAB210 |
| *Rinvoq* | AmBeed | A165008 |
| *Stat5i* | AmBeed | A303941 |
| *Bovine serum albumin* | ThermoFisher Scientific | #507533058 |
| *Trizol* | Invitrogen | #15596018 |
| *High-Capacity cDNA Reverse Transcription Kit* | ThermoFisher Scientific | #4368813 |
| *TaqMan Gene Expression master mix* | Applied biosystem | #4369016 |
| *Human IL-1 beta/IL-1F2 DuoSet ELISA* | R&D Systems | DY201 |
| *Human IL-6 DuoSet ELISA* | R&D Systems | DY206 |
| *Human CCL2/MCP-1 DuoSet ELISA* | R&D Systems | DY279 |
| *Human IL-10 DuoSet ELISA* | R&D Systems | DY217B |
| *KPL SureBlue TMB Microwell Peroxidase Substrate (1-Component)* | Sera Care | #5120-0076 |
| *PIERCE BCA protein assay kit* | ThermoFisher Scientific | #23225 |
| *ECL western blotting sub* | ThermoFisher Scientific | PI32106 |
| *Super Signal West Femto Maximum Sensitivity Substrate* | ThermoFisher Scientific | #34095 |
| *EasySep human monocytes enrichment kit* | *Stem cell technology* | *#19058* |
| *Zombie voilet* | *BioLegend* | *#77477* |
| *FITC anti-human CD14 Antibody* | *BioLegend* | *367116* |
| *APC anti-human CD86 Antibody* | *BioLegend* | *305412* |
| *L-Lactate assay kit* | *Sigma* | *MAK329-1KT* |
| *Succinate assay kit* | Sigma | MAK335-1KT |
| *Citrate assay kit* | Sigma | MAK333 |
| *Seahorse XF Real-Time ATP Rate Assay Kit* | Agilent Technologies | #103592-100 |
| *DCFDA / H2DCFDA - Cellular ROS Assay Kit* | Abcam | ab113851 |
| *VECTASHIELD Vibrance Antifade Mounting Medium with DAPI* | Vector Laboratories | H-1800 |
