# Supplementary figures and images for: Tofacitinib repairs inflammation and mitochondrial dysregulation in GM-CSF-reprogrammed RA macrophages
Source: Cell Mol Immunol. 2026 Mar 4;23(4):417–31. doi: 10.1038/s41423-026-01395-x (PMC13035809; doi:10.1038/s41423-026-01395-x)

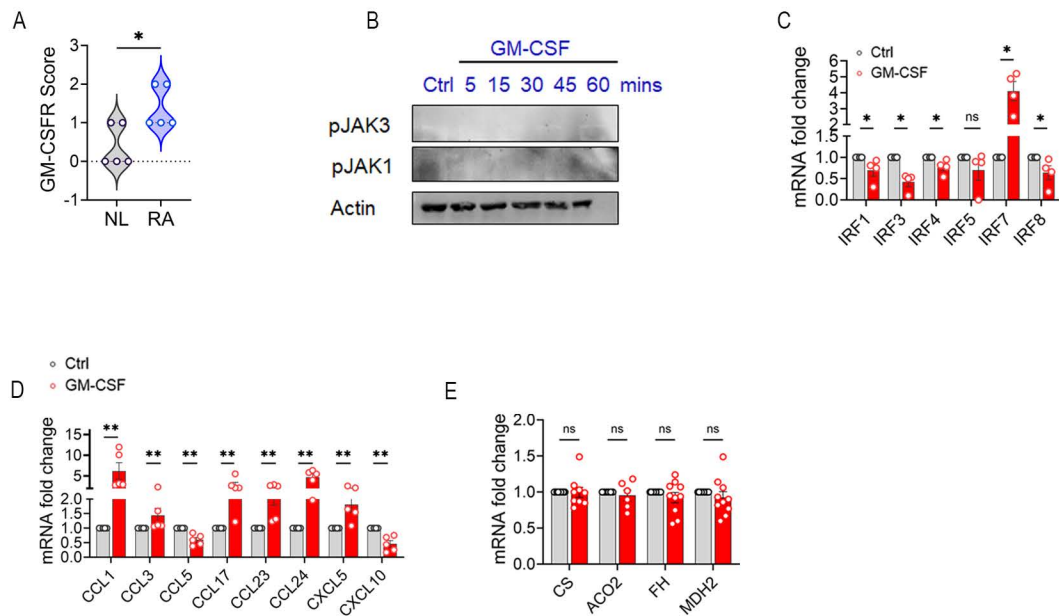

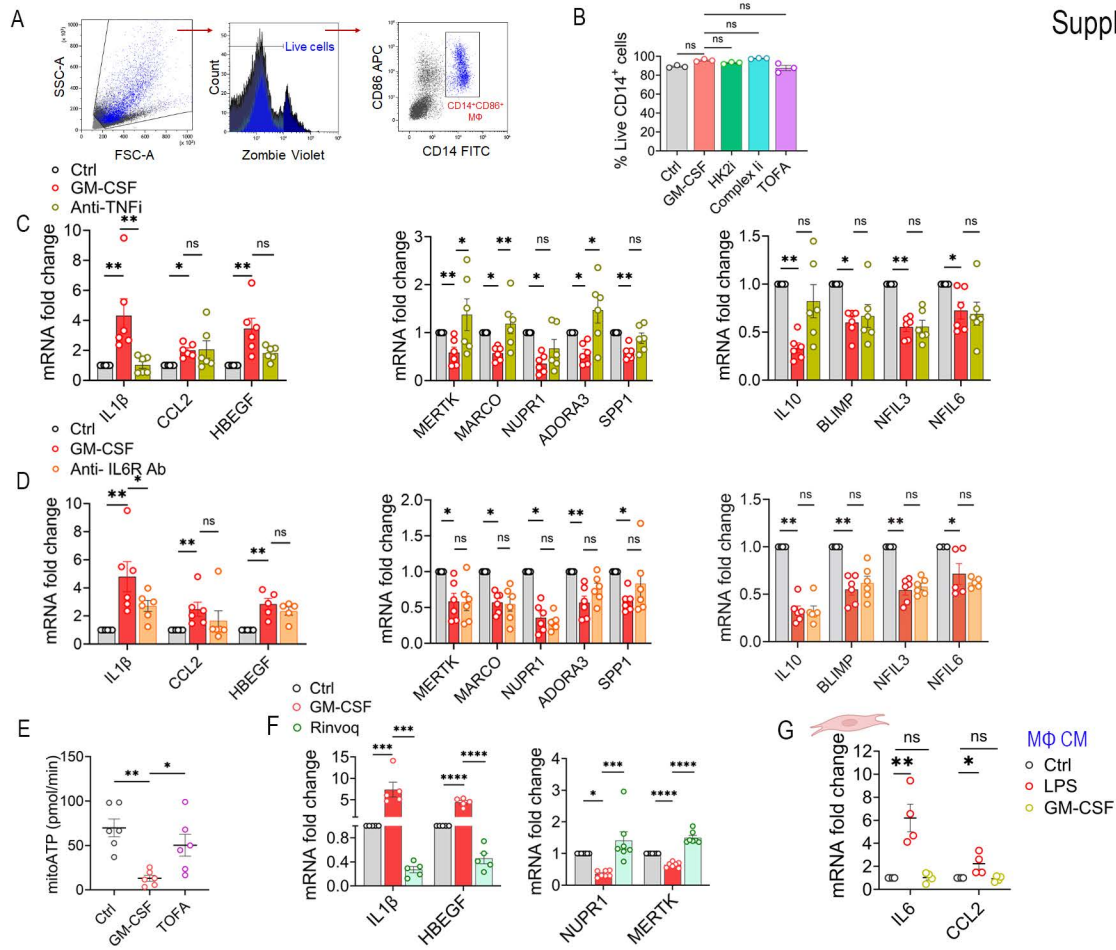

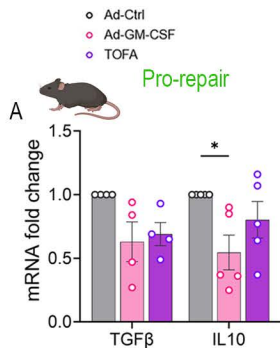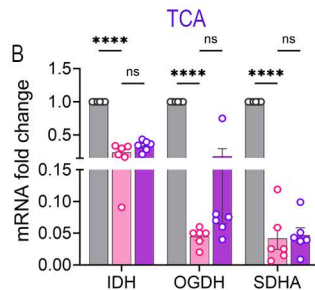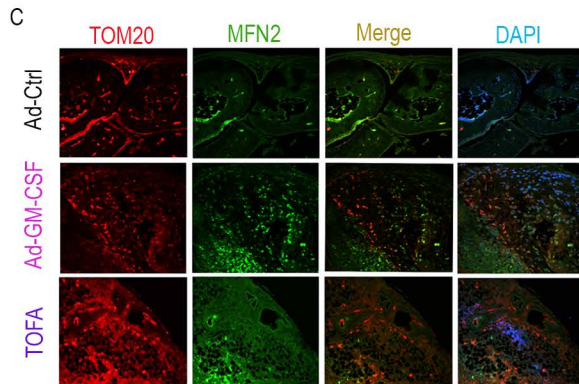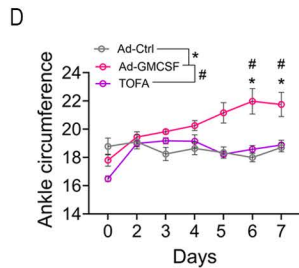

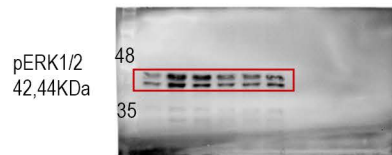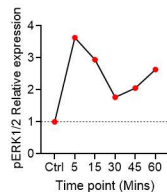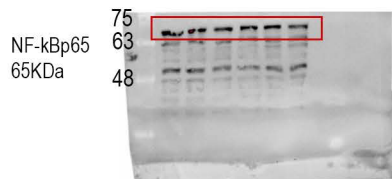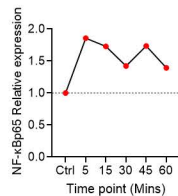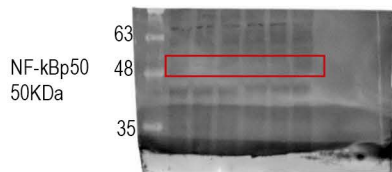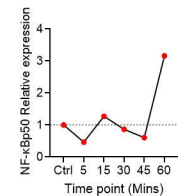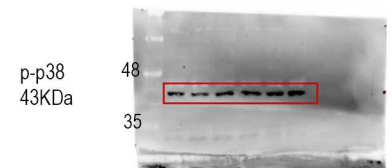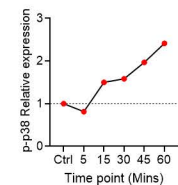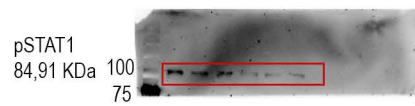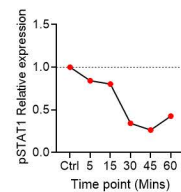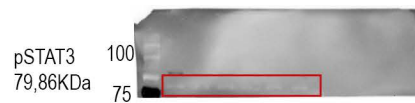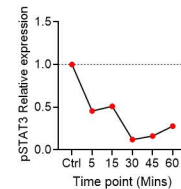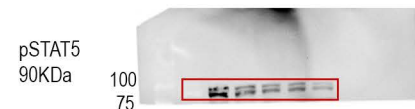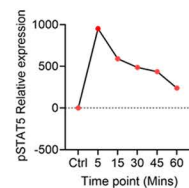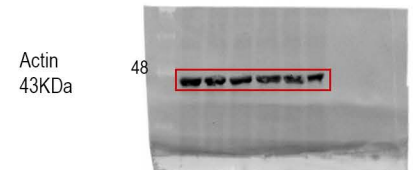

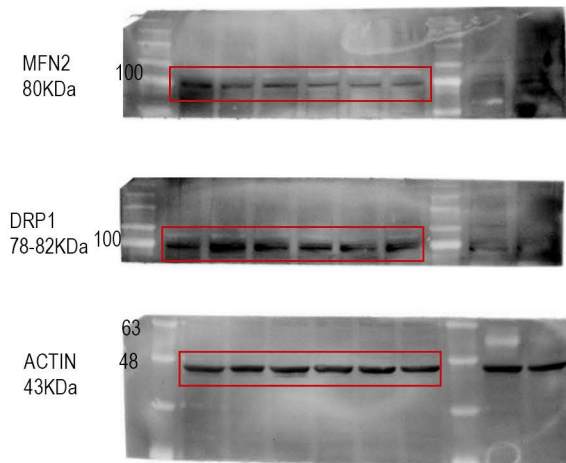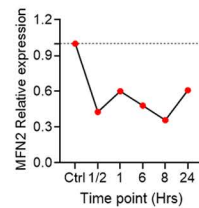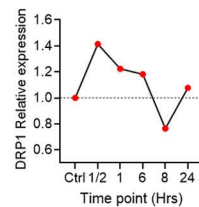

GLUT1  
45,60KDa

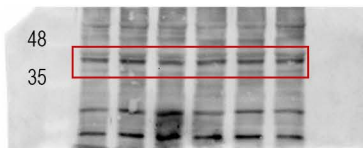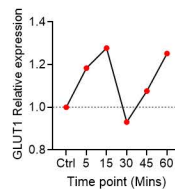

HK2  
102KDa

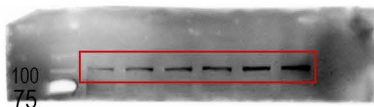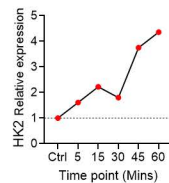

Actin  
43KDa

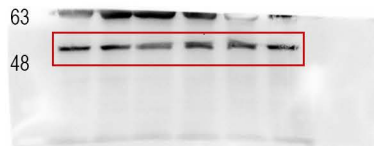

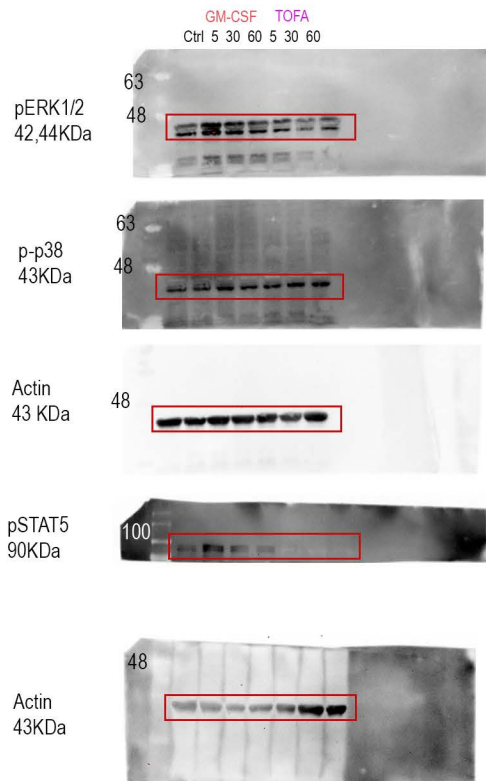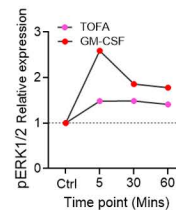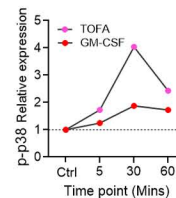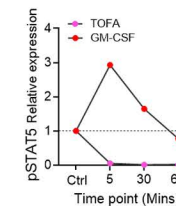

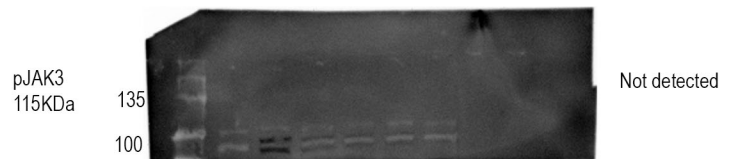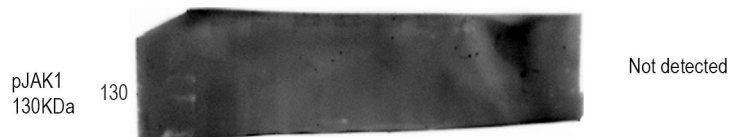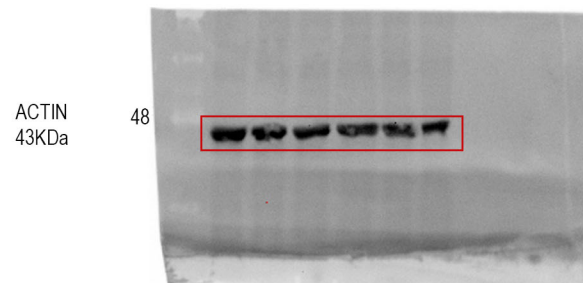

Supplement: Supplementary file 2 — Supplementary data&unprocessed images [file 41423_2026_1395_MOESM2_ESM.pdf]
