# Supplementary material for: Tofacitinib repairs inflammation and mitochondrial dysregulation in GM-CSF-reprogrammed RA macrophages
Source: Cell Mol Immunol. 2026 Mar 4;23(4):417–31. doi: 10.1038/s41423-026-01395-x (PMC13035809; doi:10.1038/s41423-026-01395-x)
Supplement: Supplementary file 3 — Supplementary figure captions [file 41423_2026_1395_MOESM3_ESM.docx]

**Supplementary Figure 1.** **Inflammation,** **signaling, and metabolic profiling of GM-MΦs.** **A** NL and RA synovial tissues were stained for GM-CSFR and scored on a 0–5 scale [79]. **B.** Myeloid cells were treated with GM-CSF (100 ng/ml) for 0–60 min, and pJAK1 and pJAK3 signaling were determined by western blot analysis, *n*=3. **C-E** RA MΦs were untreated or treated with GM-CSF for 6 h, and TFs such as IRFs (**C,** *n*=4, as shown in Fig. 2F), chemokines (**D,** *n*=5, as shown in Fig. 2G), and TCA enzymes (**E**, *n*=6-10) were detected via qRT‒PCR. Data are presented as the mean ± SEM; significant differences were determined via the Mann‒Whitney test: *p<0.05, **p<0.01, ***p<0.001, and ****p<0.0001.

**Supplementary Figure 2. Impact of anti-TNFi, anti-IL6R Ab,** **Rinvoq, or tofacitinib on GM-MΦs. A** Representative flow cytometry plots showing the gating strategy used to quantify CD14⁺CD86⁺ RA MΦs. **B** Viability of RA MΦs, untreated or treated with GM-CSF in the presence of HK2i, Complex1i, or tofacitinib for 24 h, was assessed via Zombie violet and is presented as the percentage of live CD14^+^ cells. **C‒F** RA MΦs were untreated or treated with GM-CSF (100 ng/ml) in the presence of (**C**) an anti-TNFi (10 μg/ml), (**D**) an anti-IL6R Ab (10 μg/ml), or (**F**) Rinvoq (10 μM) for 6 h, and the transcription levels of pro-inflammatory, pro-repair, and regulatory genes were quantified via qRT‒PCR, *n*=5‒7. **E** RA MΦs were untreated or treated with GM-CSF (1μg/ml) ± TOFA (100 μM), and mitochondrial (mito)ATP production was evaluated via the Seahorse XF ATP rate assay, *n*=6. **G** CM from untreated RA MΦs or those treated with LPS or GM-CSF for 24 h was used to stimulate FLS, and the levels of the IL6 and CCL2 transcripts were quantified via qRT‒PCR, n=4. The data are presented as the mean ± SEM. Statistical significance was determined by 1-way ANOVA with Tukey’s post hoc test or the Kruskal‒Wallis test with Dunn’s post hoc test. *p < 0.05, **p < 0.01, ***p < 0.001, and ****p < 0.0001.

**Supplementary Figure 3. Effect of tofacitinib on the pro-repair landscape of GM-CSF-induced arthritis.** Pro-repair gene (**A,** *n*=4-5) and TCA enzyme (**B**, *n*=6) transcription were quantified in mice that received i.a. injections of Ad-Control or Ad-GM-CSF (days 0 and 7), and the arthritic group received TOFA (10 mg/kg) daily for 10 days. **C** Ankles from Ad-Ctrl or Ad-GM-CSF ± tofacitinib were stained for MFN2 and TOM20 colocalization (magx100), *n*=3. **D** Ankle circumferences of mice that received i.a. injections of Ad-Ctrl or Ad-GM-CSF on day 0 and were treated daily with TOFA from days 2 to 7; n=10 ankles in 5 mice. Data are presented as mean ± SEM; significant differences were determined via 2-way ANOVA with Tukey’s method for adjusting for multiple comparisons: *p<0.05, **p<0.01, ***p<0.001, and ****p<0.0001.

**Supplementary Figure 4.** Western blot data were quantified via an iBright 1500. The signal intensities were normalized to actin as a loading control, then to the baseline (0 h) to calculate the relative fold change. Supplementary data include the raw blots and corresponding quantifications for Figs. 2C, 3C, 3G, and 6B, and Suppl Fig. 1B as shown in Suppl. 4-1, 4-2, 4-3, 4-4, and 4-5, respectively.
